# The ALPK1 pathway drives the inflammatory response to *Campylobacter jejuni* in human intestinal epithelial cells

**DOI:** 10.1371/journal.ppat.1009787

**Published:** 2021-08-02

**Authors:** Jiannan Cui, Coco Duizer, Lieneke I. Bouwman, Kristel S. van Rooijen, Carlos G. P. Voogdt, Jos P. M. van Putten, Marcel R. de Zoete

**Affiliations:** 1 Department of Medical Microbiology, University Medical Center Utrecht, Utrecht, The Netherlands; 2 Department of Infectious Diseases and Immunology, Utrecht University, Utrecht, The Netherlands; University of Toronto, CANADA

## Abstract

The Gram-negative bacterium *Campylobacter jejuni* is a major cause of foodborne disease in humans. After infection, *C*. *jejuni* rapidly colonizes the mucus layer of the small and large intestine and induces a potent pro-inflammatory response characterized by the production of a large repertoire of cytokines, chemokines, and innate effector molecules, resulting in (bloody) diarrhea. The virulence mechanisms by which *C*. *jejuni* causes this intestinal response are still largely unknown. Here we show that *C*. *jejuni* releases a potent pro-inflammatory compound into its environment, which activates an NF-κB-mediated pro-inflammatory response including the induction of *CXCL8*, *CXCL2*, *TNFAIP2* and *PTGS2*. This response was dependent on a functional ALPK1 receptor and independent of Toll-like Receptor and Nod-like Receptor signaling. Chemical characterization, inactivation of the heptose-biosynthesis pathway by the deletion of the *hldE* gene and *in vitro* engineering identified the released factor as the LOS-intermediate ADP-heptose and/or related heptose phosphates. During *C*. *jejuni* infection of intestinal cells, the ALPK1-NF-κB axis was potently activated by released heptose metabolites without the need for a type III or type IV injection machinery. Our results classify ADP-heptose and/or related heptose phosphates as a major virulence factor of *C*. *jejuni* that may play an important role during *Campylobacter* infection in humans.

## Author summary

Understanding the virulence mechanism of pathogenic bacteria is an important step in the development of targeted intervention or treatment strategies. *Campylobacter jejuni* is the most common cause of bacterial foodborne enteritis, but the underlying molecular cause of *C*. *jejuni*-mediated disease remains unresolved. In this study, we identify ADP-heptose and/or related heptose phosphates, released by *C*. *jejuni* into its environment during growth, as a novel potent *C*. *jejuni* activator of inflammation in intestinal epithelial cells. *C*. *jejuni*-derived heptose phosphates signals through the intracellular receptor ALPK1 and induces a wide range of pro-inflammatory genes. Revealing the importance of the ALPK1 signaling axis during *C*. *jejuni* infection may pave the road for the development of new therapeutic strategies that target this inflammatory pathway or the production and release of ADP-heptose and/or related heptose phosphates by *Campylobacter*.

## Introduction

Mucosal surfaces face the continuous challenge of accommodating beneficial microbiota while preventing infection by bacterial pathogens. The difficulty of this task is illustrated by the estimated 3–5 billion cases of gastro-intestinal illness each year [1]. One of the leading causes of human enterocolitis is the bacterial pathogen *Campylobacter jejuni*. Human campylobacteriosis manifests as mild to severe intestinal discomfort with abdominal pain, fever, and (bloody) diarrhea. In about one percent of the cases, serious complications may follow such as the acute auto-immune paralyzing neuropathy Guillain-Barré syndrome [2]. Despite its large health impact and extensive research efforts, the molecular basis of *Campylobacter* pathogenesis is still largely unresolved. Once ingested, *C*. *jejuni* crosses the mucus layer and colonizes the bottom of the crypts of the colon. This is believed to be followed by breaching of the epithelial layer and triggering of immune responses that lead to a massive neutrophil influx with crypt abscess formation [3–5], which likely explains an important part of the acute inflammatory pathology observed during *C*. *jejuni* infection.

A key feature in *C*. *jejuni* pathogenesis therefore appears to be the interaction with immune receptors on epithelial and innate immune cells and the subsequent induction of a potent innate immune response. In the appropriate *in vitro* environment *C*. *jejuni* adheres to and invades epithelial cells, and is rapidly ingested by professional phagocytes [4,6–11]. The potent inflammatory response that accompanies intestinal barrier breach is assumed to result from interaction of bacterial cell wall constituents with multiple types of innate immune receptors. Indeed, various studies have shown that *C*. *jejuni* is able to activate Toll-like receptors TLR1, TLR2, TLR4, TLR6 (but not TLR5 and TLR9) [12], the intracellular peptidoglycan receptors NOD1 and NOD2 [13–15], and the NLRP3 inflammasome [16]. The vast majority of the different signaling cascades initiated by the *C*. *jejuni* innate receptor recognition then leads to the activation of the transcription factor nuclear factor kappa-B (NF-κB), which is a master regulator of immune and inflammatory genes [17].

While *C*. *jejuni* clearly possesses the classical “pathogen-associated molecular patterns” (PAMPs) that can drive NF-κB responses in isolated *in vitro* cell systems, there is growing awareness that these molecules, which are typically present in both bacterial pathogens and harmless bacterial commensal species, may play a more general role in intestinal immune homeostasis [17]. This led us to search for the existence of novel activators of NF-κB from *C*. *jejuni* that may contribute to the establishment of human infection. Here, we provide evidence that *C*. *jejuni* induces cellular inflammation in HeLa 57A cells and intestinal cells mainly by the release of the potent pro-inflammatory nucleotide sugar ADP-heptose and/or related heptose phosphates into its microenvironment. These inflammatory metabolites enter the human host cell and activate the NF-κB pathway in an alpha-kinase 1 (ALPK1)-dependent fashion. The ALPK1-NF-κB signaling axis activated during *C*. *jejuni* infection may contribute significantly to the pathogenesis of campylobacteriosis.

## Results

### *Campylobacter* conditioned media contain a proinflammatory factor that potently activates NF-κB independently of TLRs and NOD1/NOD2

**Fig 1:**
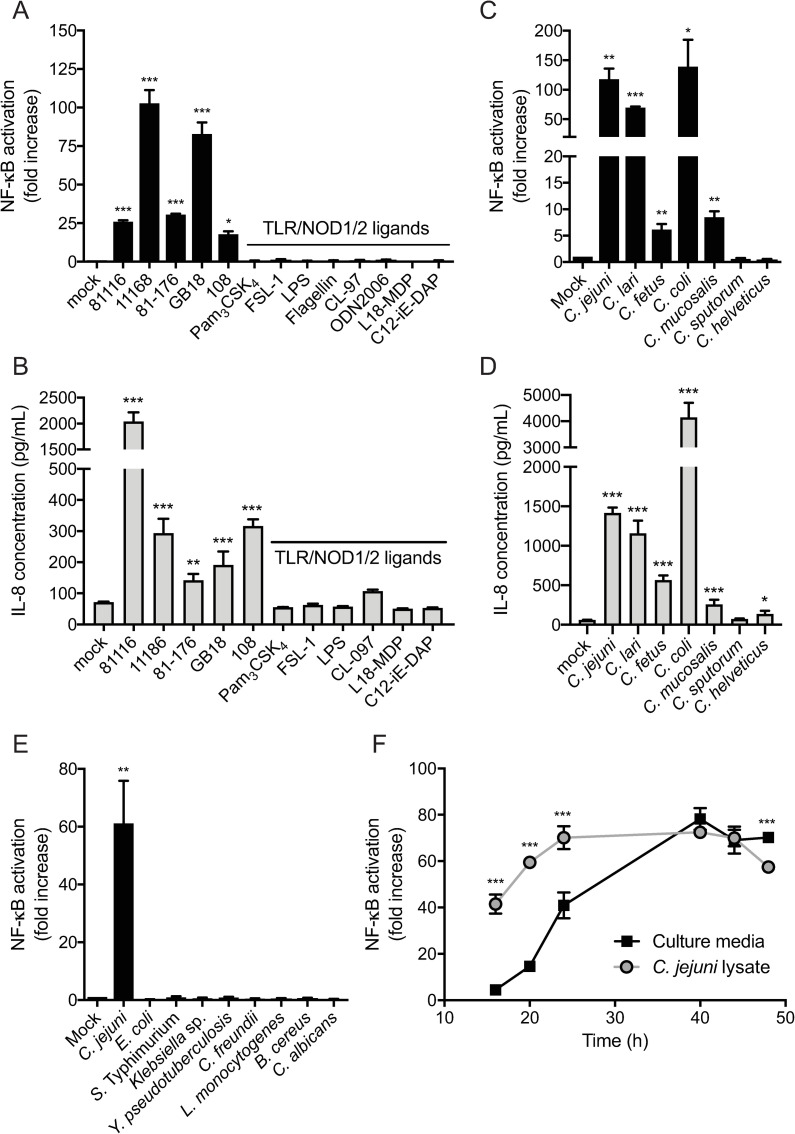
*Campylobacter* releases a novel activator of NF-κB into the culture supernatant. (A, B) HeLa 57A cells were stimulated with sterile conditioned culture supernatant of five different *C*. *jejuni* strains or with the TLR/NOD1/2 ligands Pam_3_CSK_4_, FSL-1, Poly(I:C), LPS, flagellin, CL-97, ODN2006, L18-MDP or C12-iE-DAP. (C-E) Sterile conditioned culture supernatants of *C*. *jejuni*, *C*. *lari*, *C*. *fetus*, *C*. *coli*, *C*. *mucosalis*, *C*. *sputorum* and *C*. *helveticus*, *E*. *coli*, *S*. Typhimurium, *Klebsiell*a sp., *Y*. *pseudotuberculosis*, *C*. *freundii*, *L*. *monocytogenes*, *B*. *cereus* or *C*. *albicans* were used to stimulate HeLa 57A cells for 5 h (NF-κB) or 18 h (IL-8). (F) HeLa 57A cells were stimulated with sterile *C*. *jejuni* strain 81116-conditioned culture supernatant or *C*. *jejuni* strain 81116 cells disrupted via sonification at timepoints 16, 20, 24, 40, 44 and 48 h after the start of culturing. Asterisks refer to statistically significant differences between NF-κB responses from culture media and *C*. *jejuni* lysates. *C*. *jejuni* was cultured in HI broth. NF-κB activation is measured as relative luciferase units and presented as fold increase in stimulated versus unstimulated cells. IL-8 secretion is depicted as pg/ml after correcting for background. Values represent the mean ± SEM of three independent experiments performed in duplicate. *p < 0.05, **p < 0.01, and ***p < 0.001.

In search of novel immune stimulating factors, *C*. *jejuni* strain 81116 culture supernatant was screened for NF-κB activating capacity using HeLa 57A cells carrying an NF-κB-driven luciferase reporter gene. Stimulation of these reporter cells with *C*. *jejuni* conditioned culture media for 5 h strongly activated NF-κB (Fig 1A) and induced the secretion of IL-8 (Fig 1B) in contrast to unconditioned media. Conditioned media from four other *C*. *jejuni* strains isolated from various sources (e.g. human, chicken) similarly activated NF-κB and induced IL-8 secretion (Fig 1A and 1B and S1 Table), although the strength of the response varied between strains. As NF-κB activation often follows the recognition of bacterial PAMPs by TLRs or NOD1 and NOD2, we determined the functional TLR and NOD receptor repertoire of HeLa 57A cells. Stimulation of the cells with the TLR agonists Pam_3_CSK_4_, LTA, Poly I:C, LPS, flagellin, CL-97, or ODN2006 did not induce NF-κB activation or IL-8 secretion, indicating the absence of a functional TLR1, TLR2, TLR3, TLR4, TLR5, TLR6, TLR7, and TLR9 signaling pathway. Similarly, the NOD1 and NOD2 ligands C12-iE-dap and L18-MDP yielded no inflammatory response in these cells (Fig 1A and 1B). Of a selection of six other *Campylobacter* species (*C*. *lari*, *C*. *fetus*, *C*. *coli*, *C*. *mucosalis*, *C*. *sputorum* and *C*. *helveticus*), four were also able to activate NF-κB and induce the secretion of IL-8, suggesting that the immunostimulatory factor is relatively conserved within the genus *Campylobacter* (Fig 1C and 1D). A collection of other clinically important enteropathogens, including *Salmonella* Typhimurium, *Yersinia pseudotuberculosis* and *Listeria monocytogenes*, was also examined for the ability to secrete similar immunostimulatory factors but did not exhibit any detectable NF-κB activation in the HeLa 57A reporter cells (Fig 1E). Combined, these results indicate that *C*. *jejuni* conditioned media contain a previously unrecognized immunostimulator, absent from the medium of a range of other well-known enteropathogens, that potently activates NF-κB via a TLR- and NOD1/2-independent mechanism.

### *C*. *jejuni* releases the proinflammatory factor in a growth phase dependent manner

To learn more about the origin and release of the immunostimulatory factor from *C*. *jejuni* over time, lysed bacterial cells and culture supernatants were collected at different growth phases and tested for their potency to activate NF-κB (Figs 1F and S1). Bacterial lysate from early log phase strongly activated NF-κB, suggesting that the immunostimulatory factor is either in or associated with the bacterial cells at this stage. Conversely, NF-κB stimulating activity in the medium was low in early log phase and maximal in stationary phase grown *C*. *jejuni* (Fig 1F), suggesting gradual release of the compound from the bacteria into the medium after cessation of exponential growth. Similar results were obtained with *C*. *jejuni* strains 11168, 81–176, GB18 and 108 in two additional *C*. *jejuni* culture media, suggesting NF-κB-activation dynamics are conserved within the *C*. *jejuni* species and not dependent on the composition of the growth media (S2 Fig)

### Chemical characterization identifies the proinflammatory factor as a small glycan

**Fig 2:**
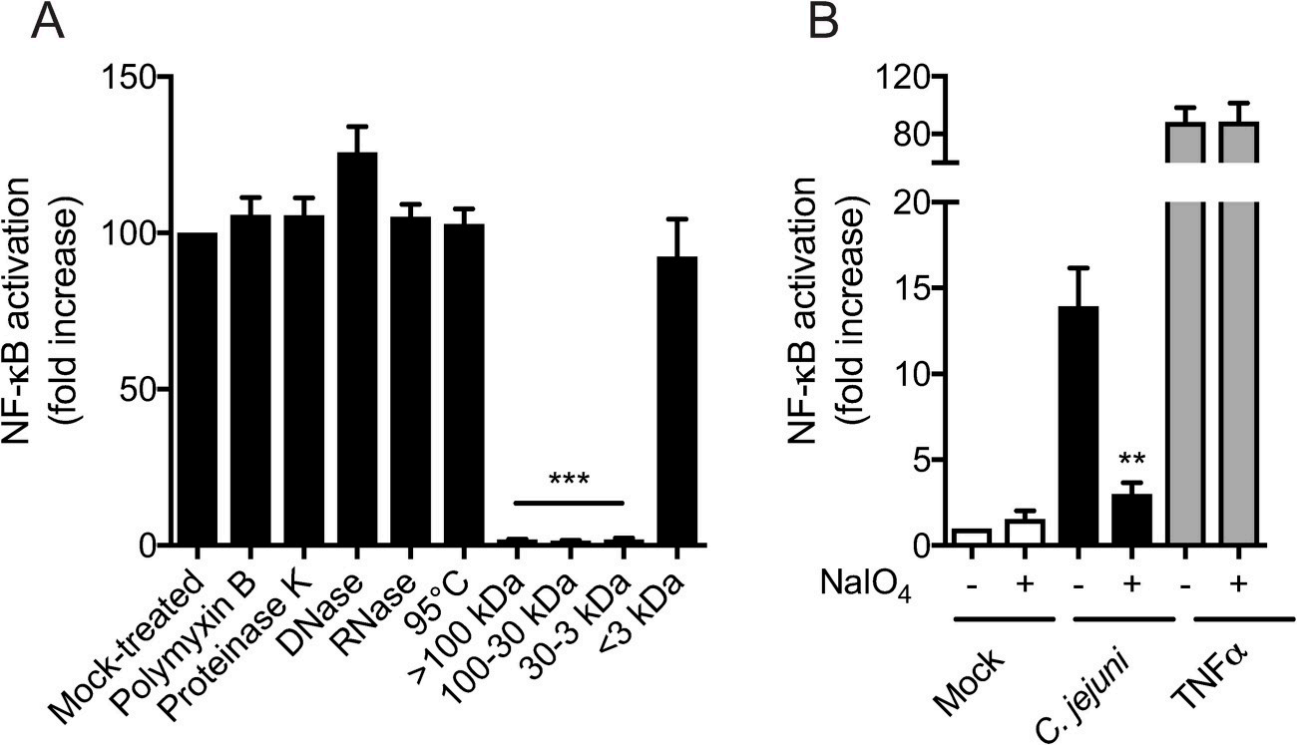
Molecular characterization of the *C*. *jejuni*-released proinflammatory factor. (A) HeLa 57A cells were stimulated with sterile conditioned culture medium that was either mock-treated, pre-treated with polymyxin B, proteinase K, DNase, or RNase at 37°C for 1 h, incubated at 95°C for 10 min, or separated based on size using 100 kDa, 30 kDa and 3 kDa cut-off filters prior to cell stimulation. (B) *C*. *jejuni* conditioned culture medium or TNFα were treated with 10 mM sodium periodate (NaIO_4_) or mock-treated prior to stimulation of HeLa 57A cells. NF-κB activation is measured as relative luciferase units and presented as fold increase in stimulated versus unstimulated cells. Values represent the mean ± SEM of three independent experiments performed in duplicate. *p < 0.05, **p < 0.01, and ***p < 0.001.

In search of the nature of the *C*. *jejuni* NF-κB inducing factor, the conditioned culture supernatant was subjected to a series of analytical assays. Incubation with polymyxin B, proteinase K, DNase and RNase did not significantly reduce the ability to activate NF-κB (Fig 2A), suggesting that the functional domain of the factor is not lipid A, of proteinaceous nature, DNA or RNA, respectively. Similarly, exposure to 95°C for 10 min did not reduce NF-κB activity, indicating considerable heat stability of the compound (Fig 2A). This characteristic may also be reflected by the sustained activity of the factor in culture medium incubated for multiple days (Fig 1F). Size exclusion analysis using an array of size-exclusion filters indicated that the factor is smaller than 3 kDa (Fig 2A). Combined with the solubility, these characteristics may suggest that the factor is (partially) of glycan nature. Therefore, the *C*. *jejuni* conditioned media was treated with sodium periodate, which oxidizes mono- and oligosaccharides. Sodium periodate treatment completely abolished NF-κB activation by the *C*. *jejuni* culture supernatant, while not affecting NF-κB signaling by TNF-α (Fig 2B). Overall, these results indicate that the induction of proinflammatory activity by the *C*. *jejuni* released factor is mediated by either a small, soluble, heat-stable glycan or glycan conjugate.

### The *C*. *jejuni* heptose biosynthesis pathway is involved in NF-κB activation

**Fig 3:**
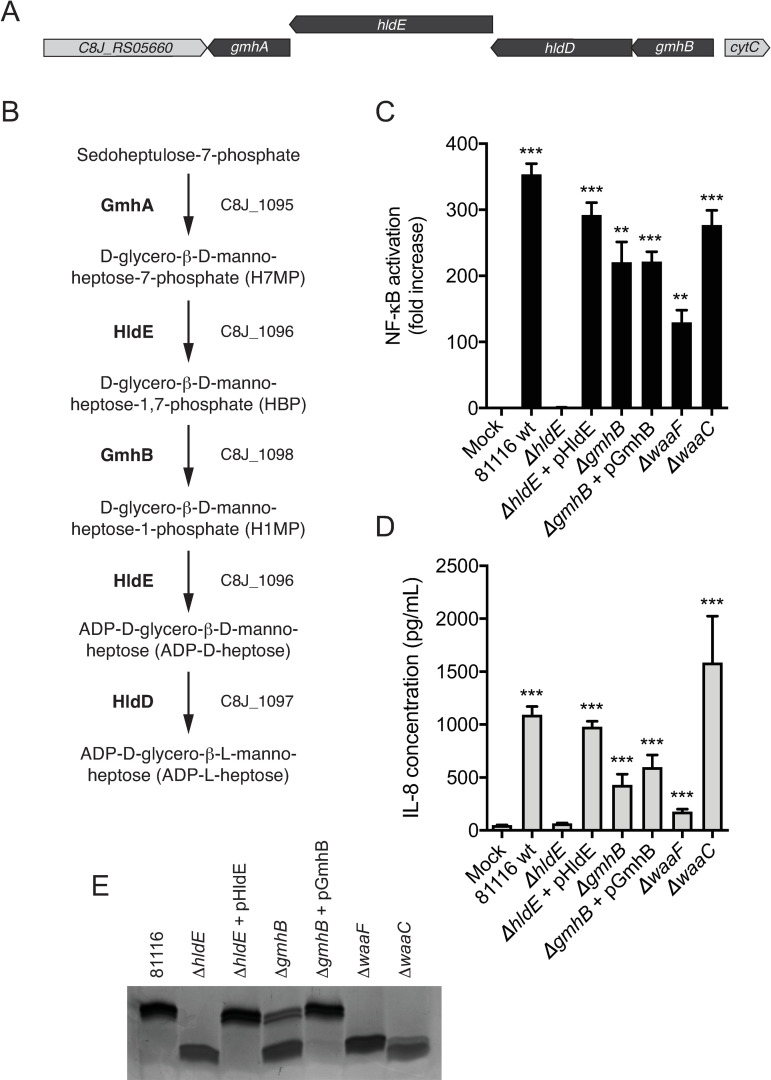
The heptose biosynthesis pathway is involved in *C*. *jejuni*-mediated NF-κB activation. (A) Schematic depiction of the heptose biosynthesis gene cluster (in black) in the genome of *C*. *jejuni* 81116. (B) The putative heptose biosynthesis enzymatic pathway of *C*. *jejuni*. Depicted are the metabolic intermediates, with enzyme names (in bold) and gene names as annotated for *C*. *jejuni* 81116. (C, D) HeLa 57A cells were stimulated with sterile conditioned culture medium of wild type *C*. *jejuni* 81116 or a selection of (complemented) mutant strains. NF-κB activation is measured as relative luciferase units and presented as fold increase in stimulated versus unstimulated cells. IL-8 secretion is depicted as pg/ml after correcting for background. Values are presented as the mean ± SEM of three independent experiments performed in duplicate. *p < 0.05, **p < 0.01, and ***p < 0.001. (E) Silverstained SDS-PAGE of LOS derived from wild type *C*. *jejuni* 81116 or the indicated (complemented) mutant strains.

Recently, it was shown that the Gram-negative bacterial metabolites heptose biphosphate (HBP) and ADP-heptose can be sensed by mammalian host cells through the newly identified intracellular ALPK1, which results in NF-κB-mediated inflammatory responses [18–20]. The vast majority of Gram-negative bacteria produce these metabolites as part of the heptose biosynthesis pathway, which is required for lipopolysaccharide (LPS) synthesis. However, heptose metabolites are usually effectively retained within the bacterial cell and enters the host cell only via direct injection by specialized bacterial secretion systems [19,21–24], which are not known to be present in *C*. *jejuni*. Examination of the *C*. *jejuni* 81116 genome revealed, as expected, the presence of a complete heptose biosynthesis pathway (Fig 3A). *C*. *jejuni* utilizes the dual-enzyme *hldE* that contains both kinase and ADP transferase activity and catalyzes the second as well as the fourth step in the synthesis pathway (Fig 3B), combining the activity of the *hldA* and *hldC* genes that are present in several other bacterial species including *Neisseria meningitidis* [18].

To examine if the heptose biosynthesis pathway is involved in the production of the *C*. *jejuni* proinflammatory factor, *hldE*, a crucial gene within the pathway (Fig 3B), was selectively deleted from *C*. *jejuni* through homologous recombination. Inactivation of *hldE* in other bacteria is reported to results in a break in the heptose biosynthesis pathway [19] and accumulation of heptose-7-phosphate that is no longer converted into HBP or ADP-heptose. When a functional copy of *hldE* was deleted from the genome of *C*. *jejuni* 81116, the conditioned culture medium was completely devoid of NF-κB-stimulating activity in HeLa 57A cells, whereas genetic complementation of the mutant strain with *hldE* expressed from a shuttle vector restored NF-κB activation and IL-8 secretion to wild type levels (Fig 3C and 3D). Analysis of the *C*. *jejuni* lipooligosaccharide (LOS) from the 81116Δ*hldE* mutant showed an LOS of markedly reduced size as compared to the wild type *C*. *jejuni* 81116 (Fig 3E) [25]. Similar to the NF-κB activation, the LOS phenotype reverted to wild type size in the 81116Δ*hldE* mutant strain expressing *hldE* from a shuttle vector. The results show that NF-κB activation induced by the conditioned culture supernatant of *C*. *jejuni* requires a functional *hldE* gene. Since the active heptose metabolite activates NF-κB without being artificially introduced into the host cell by transfection, electroporation or permeabilization [19,20] and since ADP-heptose is the predominant heptose metabolite in bacterial cells [20], the most likely bioactive candidate in *C*. *jejuni* culture supernatant is ADP-heptose. However, we cannot exclude a contribution of related heptose phosphates.

The gene encoding for D-glycero-beta-D-manno-heptose-1,7-bisphosphate 7-phosphatase, *gmhB*, is the third gene in the heptose biosynthesis pathway and predicted to remove the 7-phosphate from heptose-1,7-biphosphate to allow for the catalysation of the ADP transfer resulting in ADP-heptose. Deletion of *gmhB* in *C*. *jejuni* 81116 did not result in significant reduction of NF-κB stimulation by conditioned culture supernatant (Fig 3C and 3D). When examining the LOS of 81116Δ*gmhB*, size analysis showed that a substantial part of the LOS remained of wild type size, suggesting that in the absence of *gmhB*, *C*. *jejuni* can still produce a full-length LOS chain (Fig 3E). Deletion of two additional genes, *waaF* and *waaC*, that act downstream of the synthesis of ADP-heptose by catalysing the transfer of heptose onto the LOS, did not abolish NF-κB activation. The LOS in these two mutants was found to be truncated due to loss of heptoses and additional LOS glycans.

### *In vitro* synthesized ADP-heptose using recombinant *C*. *jejuni* enzymes activates NF-κB

**Fig 4:**
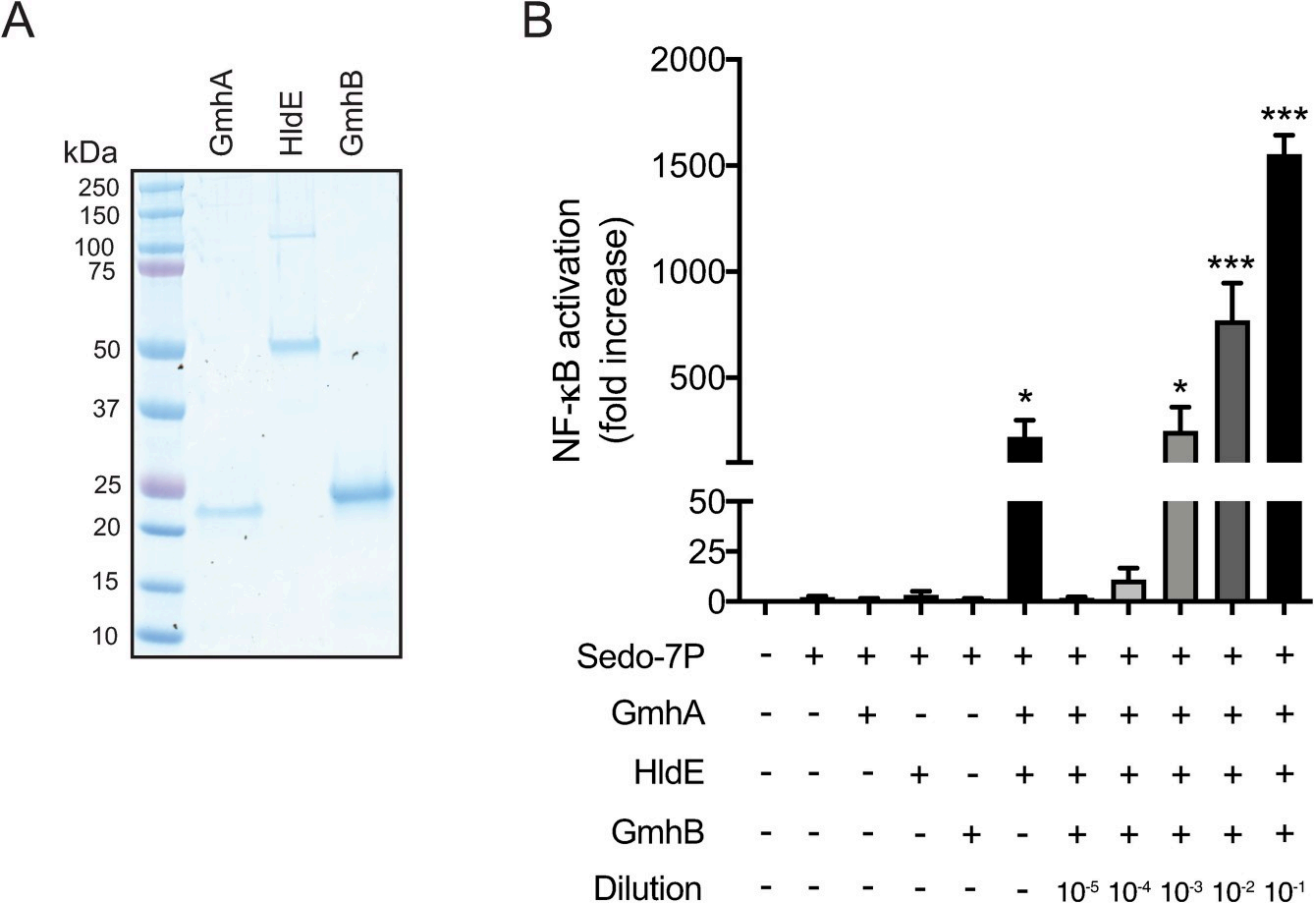
*In vitro* synthesized heptose metabolites prepared using *C*. *jejuni* enzymes activate NF-κB. (A) SDS-PAGE gel stained with Coomassie Blue to visualize the purified His-tagged *C*. *jejuni* proteins GmhA, HldE and GmhB. (B) Inclusion of individual enzymes together with sedoheptulose-7-phosphate did not result in measurable NF-κB activity, whereas the combination of GmhA and HldE, and GmhA, HldE and GmhB yielded in NF-κB activating compounds. NF-κB activation is measured as relative luciferase units and presented as fold increase in stimulated versus unstimulated cells. Values represent the mean ± SEM of three independent experiments performed in duplicate. *p < 0.05, **p < 0.01, and ***p < 0.001.

To gain further evidence that ADP-heptose is responsible for the NF-κB activation by conditioned culture medium from *C*. *jejuni*, we expressed *C*. *jejuni* GmhA, HldE and GmhB as recombinant proteins in *E*. *coli* (Fig 4A). The enzymes were used to enzymatically synthesize ADP-heptose by using sedoheptulose-7-phosphate as a starting product. As predicted, both sedoheptulose-7-phosphate and heptose-7-phosphate, the product of GmhA, were not able to induce NF-κB activation in HeLa 57A cells (Fig 4B). Similarly, inclusion of only HldE or GmhB in the reaction mixture did not result in a proinflammatory endproduct. When the first two enzymes in the pathway, GmhA and HldE where combined, the resulting product—either HBP or ADP-heptose-7P [18,19]—was able to induce a moderate NF-κB response. When all three enzymes of the heptose synthesis pathway that catalyze the pathway up to the production of ADP-D-heptose (GmhA, HldE and GmhB) were combined in the synthesis reaction, potent NF-κB activation in HeLa 57A cells was observed, roughly 1000-fold more potent as seen with GmhA and HldE alone. This highlights that likely *C*. *jejuni* ADP-heptose, the predicted product from the enzymatic reaction with *C*. *jejuni* GmhA, HldE and GmhB, is the strongest activator of NF-κB, although HBP and/or ADP-heptose-7P could also contribute to a lesser extent.

### *C*. *jejuni*-derived heptose phosphates activate NF-κB through ALPK1

**Fig 5:**
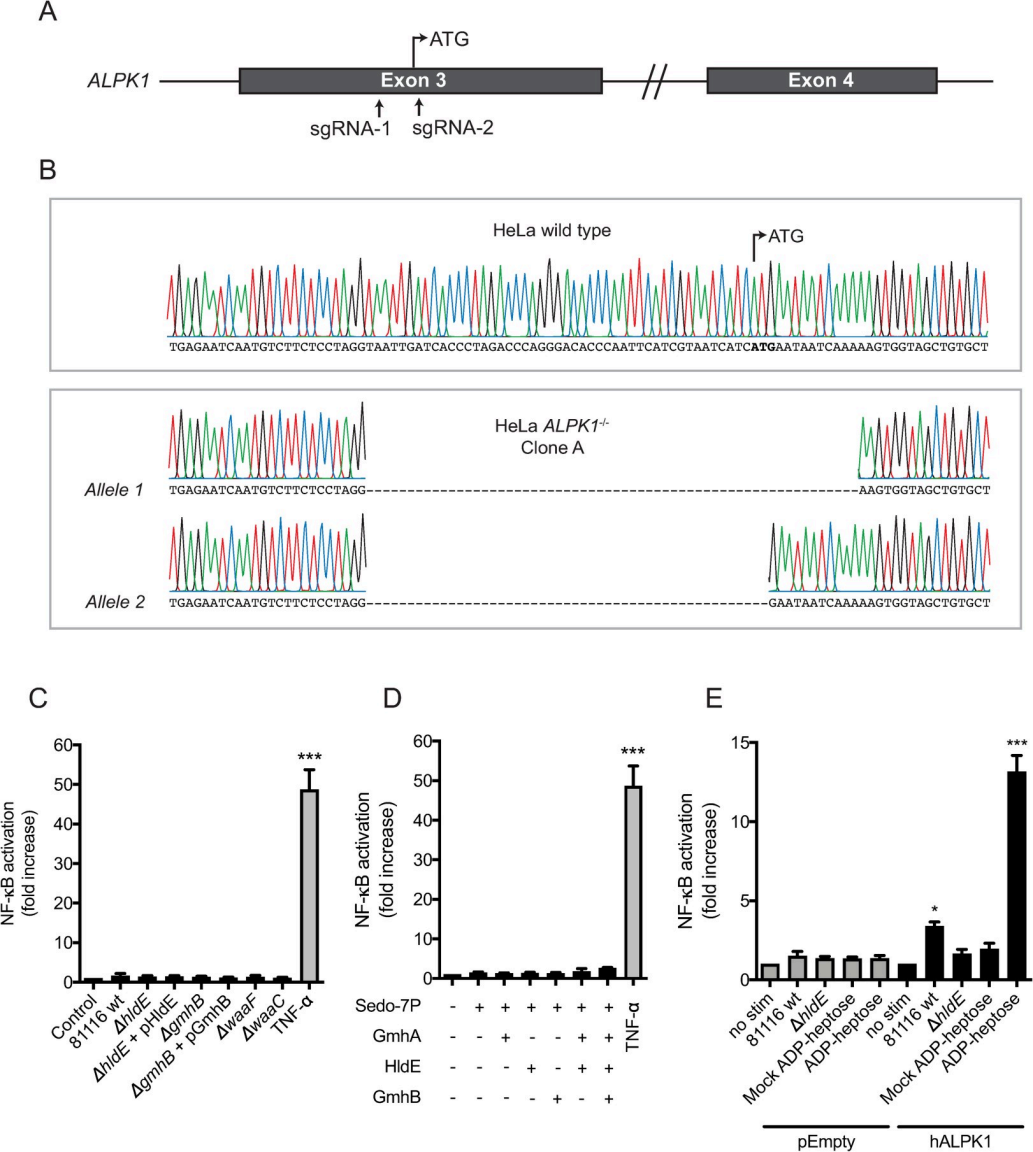
*C*. *jejuni*-derived ADP-heptose and/or heptose metabolites-mediated NF-κB activation is dependent on ALPK1. (A) Schematic overview of the CRISPR/Cas9 deletion strategy, showing the locations of the two sgRNAs targeting the *ALPK1* exon 3. (B) Sequence analysis of the *ALPK1* exon 3 from wild type HeLa 57A cells and to HeLa 57A ALPK1^-/-^ Clone A. HeLa 57A cells deficient in ALPK1 (HeLa 57A ALPK1^-/-^, Clone A) were stimulated for 5 h with (C) sterile conditioned culture medium of wild type *C*. *jejuni* 81116 or the indicated (complemented) mutant strains, or (D) with the endproduct of various heptose synthesis reactions. As a positive control, TNFα was included. (C) HeLa 57A ALPK1^-/-^ cells (Clone A) were transfected with an empty expression plasmid or transfected with a plasmid expressing human *ALPK1*, and subsequently stimulated for 5 h with sterile conditioned culture medium of wild type *C*. *jejuni* 81116, *C*. *jejuni* 81116Δ*hldE*, mock ADP-heptose of ADP-heptose derived from *in vitro* synthesis using *C*. *jejuni* enzymes. NF-κB activation was measured as relative luciferase units and presented as fold increase in stimulated versus unstimulated cells. Results are the mean ± SEM of three independent experiments performed in duplicate. *p < 0.05, **p < 0.01, and ***p < 0.001.

ADP-heptose activates ALPK1 intracellularly, leading to phosphorylation of tumor necrosis factor receptors-associated factor (TRAF)-interacting protein with forkhead-associated domain (TIFA) and subsequent NF-κB activation [19]. Both *ALPK1* and *TIFA* are expressed in HeLa 57A cells, as determined by RT-PCR (S3 Fig) To provide further evidence that C. *jejuni* activates this inflammatory pathway, ALPK1 deficient HeLa 57A cells (independent clone A and B) were constructed using CRISPR/Cas9-mediated targeted mutagenesis (Figs 5A, 5B and S4). When ALPK1^-/-^ HeLa 57A cells were stimulated with the various wild type and mutant *C*. *jejuni* strains, no NF-κB activation was observed. In contrast, stimulation of these knockout cells with TNF-α resulted in a marked NF-κB activation, showing the capacity of the cells to still respond to other inflammatory stimuli (Fig 5C). Similarly, when ALPK1^-/-^ HeLa 57A cells were stimulated with the set of *in vitro* synthesized metabolic products, none of the products were able to stimulate NF-κB response in the absence of ALPK1 (Fig 5D). When ALPK1^-/-^ HeLa 57A cells were genetically complemented by transient transfection with an ALPK1-expression plasmid, *C*. *jejuni* culture supernatant but not that of the 81116Δ*hldE* mutant regained NF-κB-stimulating activity (Figs 5E and S4), highlighting the specificity of the CRISPR/Cas9-mediated ALPK1 targeted deletion. Combined, these results strongly suggest that ALPK1 is the receptor for NF-κB-mediated inflammatory responses by *C*. *jejuni* conditioned culture medium and *C*. *jejuni*-derived ADP-heptose in HeLa 57A cells.

### C. *jejuni* heptose phosphates activate a wide set of pro-inflammatory genes

**Fig 6:**
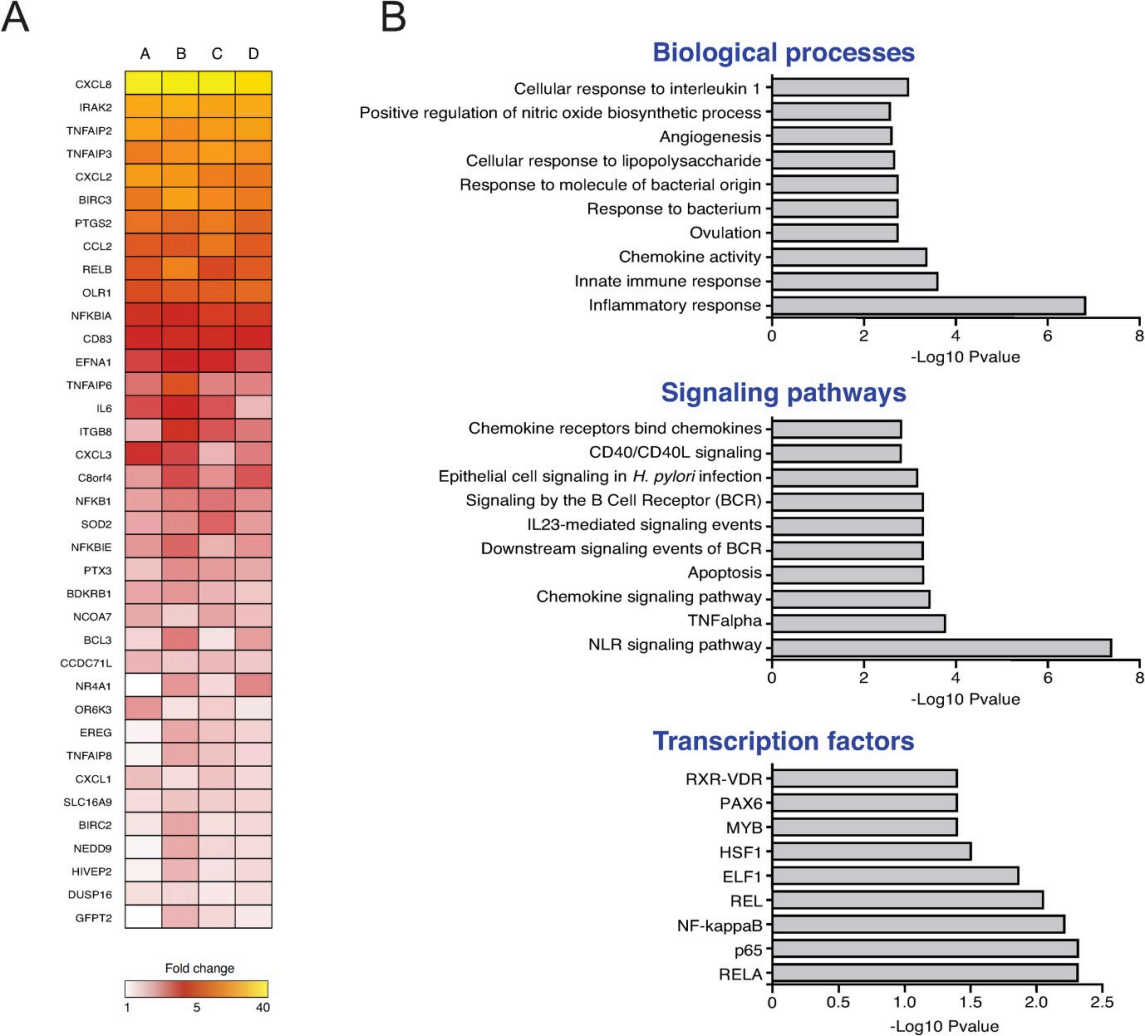
*C*. *jejuni* ADP-heptose and/or heptose metabolites activate a wide set of pro-inflammatory genes. (A) Selection of the highest upregulated genes revealed by microarray analysis of HeLa 57A cells stimulated for 2 h with sterile *C*. *jejuni* conditioned culture medium. Depicted are four independent repeats (A-D) shown as fold change over mock-stimulated conditions. (B) Analysis of the microarray data set by InnateDB on Biological process, signaling pathway or transcription factors.–LOG10 p-values represent the significance of over-representation of a process, pathway, transcription factor in the microarray data set (fold change >2 and a p-value <0.05).

NF-κB activation is a key step in the transcription of a large array of genes, including many proinflammatory immune genes. To assess the width of the immune response activated by *C*. *jejuni*-derived ADP-heptose and/or related heptose phosphates, we performed microarray analysis on HeLa 57A cells stimulated with *C*. *jejuni* conditioned culture medium. Results showed significant downregulation of 90 gene transcripts and upregulation of 237 transcripts (S2 Table). Downregulation was never more than 2-fold, while 37 out of 237 upregulated genes showed >2-fold increase in transcript levels. As is noticable in Fig 6A, all of the top ADP-heptose-induced genes are strongly associated with a proinflammatory immune program, with *CXCL8*, *TNFAIP2*, *TNFAIP3*, *CXCL2*, *CCL2* and *IL6* as major proinflammatory cytokines and chemokines. In addition, various genes involved in the initiation or modulation of inflammatory signaling, like *IRAK2*, *BIRC3*, *RELB* and *NFKBIA*, and genes involved in a number of other inflammation-related pathways (e.g. *PTGS2*, *CD83* and *ITGB8*) were also found to be significantly upregulated. Most of these genes are known to be regulated by NF-κB, suggesting that *C*. *jejuni*-mediated ALPK1 activation predominantly, if not solely, activates the NF-κB signaling pathway. Analysis of these genes using InnateDB [26] showed that the majority is connected to an inflammatory response and the innate immune system (Fig 6B). These data show that *C*. *jejuni*-derived heptose metabolites mediated NF-κB activation initiates a broad transcriptional program consisting of various proinflammatory and other immune-related genes in HeLa 57A cells.

### *C*. *jejuni*-derived heptose metabolites activate inflammatory responses in human intestinal epithelial cells

**Fig 7:**
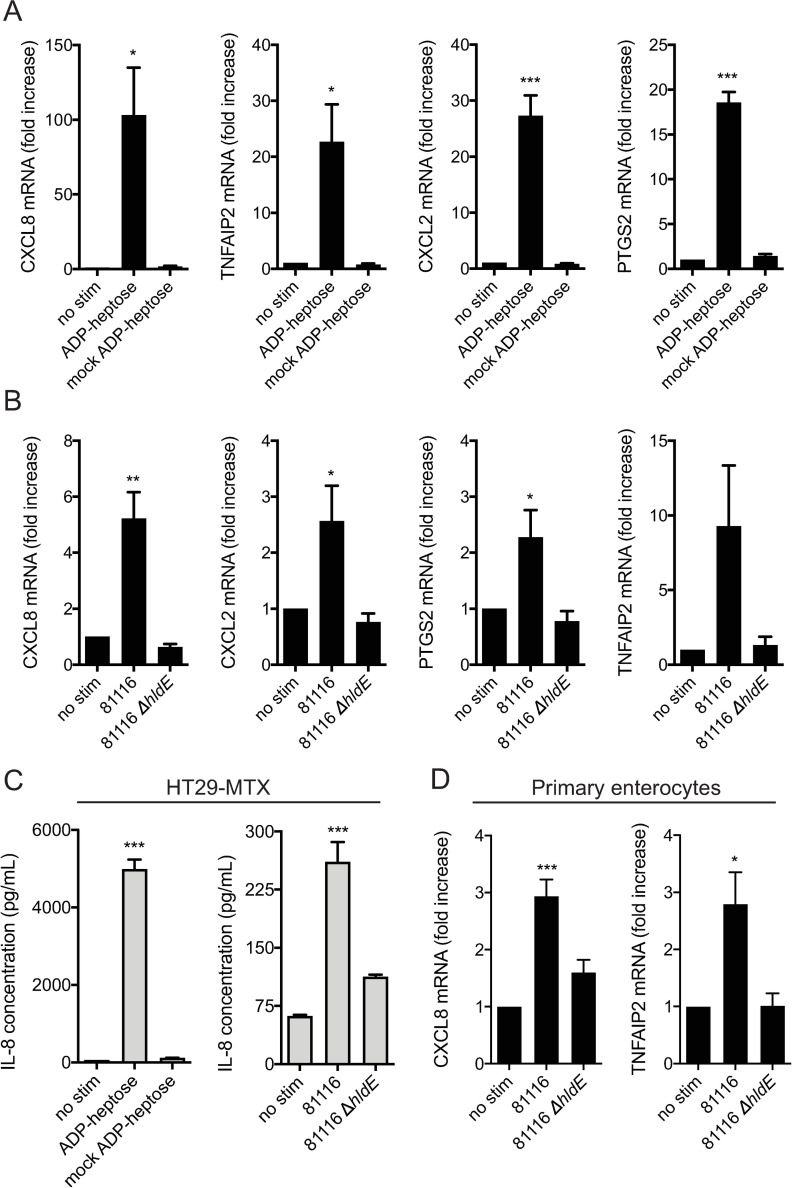
*C*. *jejuni* ADP-heptose activates inflammatory responses in human intestinal epithelial cells. Intestinal HT29-MTX cells were (A) stimulated for 3 h with *in vitro* synthesized ADP-heptose or mock reactions (mock ADP-heptose), or (B) infected for 10 h with washed viable *C*. *jejuni* 81116 or *C*. *jejuni* 81116Δ*hldE* at a multiplicity of infection of 200 after which mRNA expression of *CXCL8*, *TNFAIP2*, *CXCL2* and *PTGS2* was determined by qPCR. (C) HT29-MTX cells infected for 18 h with washed viable *C*. *jejuni* 81116 or *C*. *jejuni* 81116Δ*hldE* at a multiplicity of infection of 200 after which IL-8 concentrations were measured using ELISA. (D) *Ex-vivo* primary enterocytes were infected for 10 h with washed viable *C*. *jejuni* 81116 or *C*. *jejuni* 81116Δ*hldE* at a multiplicity of infection of 200 after which mRNA expression of *CXCL8* and *TNFAIP2* was determined by qPCR. Data are presented as fold change in transcript levels between stimulated versus unstimulated cells. IL-8 secretion is depicted as pg/ml after correcting for background. Values represent the mean ± SEM of three independent experiments performed in duplicate. *p < 0.05, **p < 0.01, and ***p < 0.001.

While HeLa 57A cells are an excellent readout cell system for identifying and analyzing novel immunostimulatory molecules, the usefulness of this cell type as a model for *C*. *jejuni* infection is clearly limited. To be able to assess whether human intestinal epithelial cells, the target cells of *C*. *jejuni* during human infection, are responsive to ADP-heptose and related heptose phosphates, HT29-MTX cells were stimulated with *in vitro* synthesized *C*. *jejuni*-derived heptose metabolites. After 3 hours of stimulation, the induction of the immune genes *CXCL8*, *TNFAIP2*, *CXCL2* and *PTGS2*, chosen based on the expression pattern seen in Fig 6 and their roles in inflammation, was evaluated using RT-PCR. Each of these genes show a significant induction after stimulation with heptose metabolites, but not after stimulation with mock-synthesized *C*. *jejuni*-derived heptose metabolites that contains all of the components of the synthesis reaction but includes heat-inactivated enzymes (Fig 7A). Similarly, HT29-MTX cells produced IL-8 after stimulation with *in vitro* synthesized heptose metabolites but not mock synthesized heptose metabolites (Fig 7C). These findings indicate that the intestinal epithelial HT29-MTX cells functionally express ALPK1, are highly sensitive to *C*. *jejuni*-derived heptose metabolites and respond by producing various inflammatory mediators.

While *in vitro* synthesized *C*. *jejuni*-derived ADP-heptose and/or related heptose phosphates and conditioned culture supernatant potently activate an inflammatory response in intestinal cells, during an infection with live bacteria other factors may play a role that may complicate the outcome. To further mimic the conditions of an intestinal *C*. *jejuni* infection and assess the involvement of heptose metabolites in the resulting enteritis, intestinal HT29-MTX cells were infected for 10 h with live *C*. *jejuni* 81116 or *C*. *jejuni* 81116Δ*hldE*, followed by assessment of inflammatory responses through RT-PCR. While wild type *C*. *jejuni* 81116 induced the transcription of all four proinflammatory genes that were evaluated (*CXCL8*, *TNFAIP2*, *CXCL2* and *PTGS2*), *C*. *jejuni* 81116Δ*hldE* did not significantly stimulate these genes (Fig 7B). Similarly, HT29-MTX cells released IL-8 after stimulation with live *C*. *jejuni* 81116 but not with *C*. *jejuni* 81116Δ*hldE* (Fig 7C). Finally, *ex-vivo* human primary enterocytes were stimulated with *C*. *jejuni* 81116 or *C*. *jejuni* 81116Δ*hldE*, which resulted in the induction of the proinflammatory genes *CXCL8* and *TNFAIP2* (Fig 7D). *CXCL2* and *PTGS2* were not upregulated in these cells. These experiments show that heptose metabolites, in the settings of an *in vitro* intestinal infection, is the major factor secreted by live *C*. *jejuni* that induces strong proinflammatory immune responses and should therefore be considered as a main driver of inflammation during human campylobacteriosis.

## Discussion

While numerous bacterial species are known to trigger host defense responses through recognition by Toll-like receptors and NOD-like receptors, the extent to which the intracellular receptor ALPK1 contributes to bacterial recognition and inflammation is only beginning to be unraveled. Here, we report for the first time that the bacterial enteropathogen *C*. *jejuni* is capable of activating ALPK1 in intestinal epithelial cells by releasing ADP-heptose into its surroundings. This release triggers the expression of a range of pro-inflammatory genes in HeLa 57A cells, cultured intestinal epithelial HT29-MTX cells and primary human enterocytes.

Colonization of the human intestinal tract by *C*. *jejuni* rapidly results in a severe intestinal inflammatory response characterized by massive neutrophil influx, epithelial damage and (bloody) diarrhea [2]. While the acute pathology resembles that of other bacterial enteropathogens, its mechanism is not as well defined. In the apparent absence of potent toxins and virulence-associated protein secretion systems, various other *C*. *jejuni* factors have been proposed to contribute to inflammation. For instance, *C*. *jejuni* has been previously shown to be able to activate TLR1/2/6 and TLR4, albeit to a very limited extent when bacteria are alive and actively growing [12]. In addition, the cytosolic peptidoglycan receptors NOD1 and NOD2, and the NLRP3 inflammasome sense the presence of *C*. *jejuni* following host cell invasion and respond by the induction of innate immune responses [13–16]. With the present identification of ADP-heptose as a potent novel inflammatory trigger, a previously unrecognized *C*. *jejuni* virulence factor that could contribute to enteritis has been discovered—despite that not all of our data was collected in intestinal cells. Identification of novel stimulators of innate immune responses is often hampered by the presence of highly potent innate immune activating contaminants such as LPS, lipopeptides, nucleotides or peptidoglycan fragments. Several lines of evidence collectively exclude a role for these ligands: (i), it was confirmed that HeLa 57A cells lack functional TLR and NOD1/2 signaling as evidenced by the absence of classical TLR and NOD1/2 agonists-induced NF-κB activation (Fig 1A and 1B and [12]); (ii), treatment of the medium with DNase, RNase, proteinase K and the lipid A neutralizing polymyxin B did not inhibit the activation of NF-κB (Fig 2A); (iii), targeted deletion of ADP-heptose producing enzymes in *C*. *jejuni* abolished the NF-κB activation (Fig 3); and (iv), genetic inactivation of the ADP-heptose receptor ALPK1 made the host cells selectively unresponsive to ADP-heptose (Fig 5).

Only a handful of other human pathogens have been described to induce ADP-heptose or HBP-mediated inflammatory responses, including *N*. *meningitidis* and *N*. *gonorrhoeae* [18], *Yersinia pseudotuberculosis* [19], *Shigella flexneri* [21,27] and *Helicobacter pylori* [22–24]. Except for *Neisseria*, all of these bacteria stimulate NF-κB through the injection of ADP-heptose directly into the cytosol of the host cell, either via a type III or type IV secretion system. Such injection systems are not present in *Campylobacter*. Instead, *Campylobacter* releases ADP-heptose into its surroundings, both during culturing and during experimental infection of intestinal cells, from which ADP-heptose enters the host cells and activates ALPK1. The release of ADP-heptose by *C*. *jejuni* showed remarkable kinetics, as it was present in bacterial lysates during the logarithmic growth phase but largely released from the bacteria into the medium after cessation of growth after ~20 hours. Noticeably, levels of intracellular ADP-heptose remained high while the concentration of extracellular ADP-heptose was rising, suggesting that *C*. *jejuni* continues to produce ADP-heptose during stationary phase (up to ~40 hours of growth). Currently, it is not yet known whether the release of ADP-heptose is an active or passive process, although the size of the molecule indicates that it likely does not diffuse through the inner and outer membrane freely. The transition from exponential bacterial growth to a cessation of growth is accompanied by major changes in bacterial metabolism and cell wall synthesis which may ultimately end in membrane instability, bacterial disintegration and death. However, under the conditions employed, *C*. *jejuni* displayed a motile, healthy spiral-shaped phenotype up to 24 hours after exponential growth. Combined, we assume that the compound is actively released from the pathogen through an as yet undefined secretion pathway. Release of ADP-heptose or related heptose metabolites is not common among other Gram-negative bacteria, as shown in Fig 1E. To date, only two other bacterial species, *N*. *meningitidis* and *N*. *gonorrhoeae*, have been shown to accumulate HBP in the culture medium. The mechanism of HBP release in these bacterial pathogens is also still unresolved. Elucidation of this mechanism, which might be shared among pathogenic *Neisseria* and *Campylobacter* species, may provide an interesting opportunity for treatment of infection. This may also shed more light on the potential release of heptose metabolites by bacterial members of the microbiota and, in line with this, whether the release or secretion of heptose metabolites provides the immune system with a strategy to distinguish between pathogenic and commensal bacterial species.

While in the current study we do not address the presence of ADP-heptose in the *C*. *jejuni* conditioned culture medium directly, several lines of evidence point towards ADP-heptose as being the responsible proinflammatory compound. First, our study shows that the *C*. *jejuni*-derived heptose metabolite does not require transfection, electroporation or digitonin-mediated permeabilization to enter the host cell, as was shown by Zhou *et al* [19] and Pfannkuch *et al* [20]. Second, ADP-heptose was shown to be the predominant heptose metabolite present within several Gram-negative bacteria, with only minor fractions of the less active HBP or d-glycero-β-d-manno-heptose 1-phosphate (H1P) [20]. In contrast, Gaudet *et al* [18] demonstrate that *Neisseria* releases HBP instead of ADP-heptose, and the heptose metabolite H1P was also shown to be able to activate ALPK1-mediated inflammatory responses, albeit while permeabilized with digitonin [28]. These findings likely show that there is variation in the type of proinflammatory heptose metabolites being secreted or injected by Gram-negative bacterial species, and highlight the need for additional research to unravel potential discrepancies.

At least four enzymatic steps are required to make biologically active ADP-heptose from sedoheptulose-7-phosphate and these are predicted to be performed by the three enzymes GmhA, HldE and GmhB in *C*. *jejuni* (Fig 3B). While GmhA alone does not synthesize a metabolic intermediate that allows for ALPK1-dependent activation of NF-κB (Fig 4B) and deletion of the second and fourth enzymatic steps in the synthesis pathway similarly abolished NF-κB activation, deletion of *gmhB* in *C*. *jejuni* did not result in loss of NF-κB stimulating ability (Fig 3C and 3D) and only a partial loss of wild type-sized LOS. Similar to our findings, deletion of *gmhB* in several other bacterial species did not lead to loss of heptose-mediated NF-κB by these bacteria [18,19]. Interestingly, deletion of *gmhB* in *H*. *pylori* resulted in a loss of NF-κB activation and cytosolic ADP-heptose in one study [20] but did not alter *H*. *pylori*’s ability to induce ALPK1/TIFA-dependent NF-κB activation in two other studies [22,23], likely reflecting strain-dependent compensatory mechanisms. The LOS from the *C*. *jejuni* 81116Δ*hldE* mutant showed an LOS of reduced size as compared to wild type *C*. *jejuni* 81116 (Fig 3E), which is consistent with the inability to incorporate heptose and additional glycans that are subsequently linked to the heptose residues shown previously [25]. Both the presence of wild type-sized LOS in *C*. *jejuni* with a deletion in *gmhB* (Fig 3E), while being only a fraction of the total LOS, and the presence of ADP-heptose-dependent autotransporter heptosylation in *Y*. *pseudotuberculosis* [19] suggests either the presence of an unidentified compensatory mechanism of 7-phosphate removal from heptose-1,7-biphosphate or a relaxed substrate specificity of the ADP transferase domain of HldE that may tolerate the presence of the 7-phosphate, resulting in ADP-heptose-7-phosphate that may be (partially) used in subsequent enzymatic steps in the pathway. In the latter scenario, free ADP-heptose-7-phosphate may be released by *C*. *jejuni* and activate ALPK1 intracellularly, as was shown possible by using synthesized ADP-heptose-7-phosphate [19]. Alternatively, heptose-1,7-biphosphate (HBP) may accumulate and be released in the *C*. *jejuni gmhB* mutant. It was shown that HBP is able to activate ALPK1 following the conversion into ADP-heptose by host enzymes intracellularly [19]. However, this process appears to require active introduction of HBP into the host cytosol using transfection with permeabilizing agents. As ALPK1 activation by the conditioned culture medium of *C*. *jejuni* Δ*gmhB* occurred without an artificial permeabilization step and the mutant still contained some seemingly fully-sized LOS species, the active compound released from this mutant strain is likely not HBP but either ADP-heptose-7-phosphate produced after bypassing the GmhB protein and a relative relaxed substrate specificity of HldE, or ADP-heptose dephosphorylated via a yet to be identified pathway.

Since the majority of pathogens that are shown to activate ALPK1 have a host range mostly restricted to humans, the relative contribution of ADP-heptose-mediated ALPK1 activation to disease has been difficult to assess beyond *in vitro* cell systems. Experimental infection of *N*. *meningitidis* in a dorsal air pouch mouse model and *Burkholderia cenocepacia* in a lung inflammation mouse model highlight the acute pro-inflammatory effects of ADP-heptose sensing [18,19]. The virulence mechanism of *C*. *jejuni* has thus far remained elusive. Our results show that infection of intestinal epithelial cells with *C*. *jejuni* yields clear ALPK1-dependent inflammatory responses (Fig 7). This classifies ADP-heptose and/or related heptose phosphates as a major virulence factor that may contribute to acute human enteritis by activating the proinflammatory ALPK1-NF-κB pathway. Our results may thus provide a major step toward understanding campylobacteriosis and open up new avenues to future therapeutic intervention or treatment strategies.

## Materials and methods

### Cell lines and culture conditions

HeLa 57A cells carrying an NF-κB luciferase reporter [29] and the human colorectal adenocarcinoma cell line HT29-MTX [30], kindly provided by Dr. Thécla Lesuffleur) were routinely cultured in 25 cm^2^ tissue culture flasks in Dulbecco’s modified Eagle medium (DMEM, Thermo Fisher) supplemented with 10% fetal calf serum (FCS) at 37°C and 10% CO_2_. Primary human intestinal epithelial cells were purchased from Lonza (CC-2931) and cultured according to the manufacturer’s instructions.

### Bacterial strains and growth conditions

All *C*. *jejuni* strains (listed in S1 Table) were routinely grown on plates containing blood agar base II (Oxoid, London, UK) and 5% horse blood (Biotrading, The Netherlands) lysed with 0.5% saponin (Sigma-Aldrich). Planktonic cultures were grown in 5 ml of HI broth (Biotrading) placed on a gyratory shaker (160 rpm) at 37°C under microaerophilic conditions (80% N_2_, 10% CO_2_, 5% O_2_, 5% H_2_). *Escherichia coli* DH5α used for cloning was grown on Luria-Bertani (LB) plates at 37°C or in 5 ml of LB broth (Biotrading) at 37°C while shaking at 160 rpm. When appropriate, culture media were supplemented with chloramphenicol (20 μg ml^-1^), kanamycin (50 μg ml^-1^) and / or ampicillin (100 μg ml^-1^). All other bacterial species were started on sheep-blood agar plates and were further cultured in BHI broth (Biotrading) at 37°C while shaking at 160 rpm.

### Preparation of *Campylobacter* supernatant and lysates

Starter cultures of *C*. *jejuni* were grown on plates for 2–3 days at 37°C under microaerophilic conditions. A pre-culture of 5 ml HI medium was subsequently inoculated with a minimal amount of starter culture and grown for 5 h at 37°C. After measuring the OD_550_, a fresh 5 ml HI culture was inoculated with the pre-culture to a starting OD_550_ of 0.001 and grown for 24 h at 37°C under microaerophilic conditions. Where required, HI medium was replaced with either Mueller Hinton broth or Bolton broth (Oxoid). The conditioned culture supernatant was collected by centrifugation at 17,000 × g for 5 min, filter-sterilized (0.22 μm pore size), and stored at –20°C until further use. Bacterial pellets were resuspended in DPBS, sonicated (3 x 5 s with 30 s pause on ice) and stored at -20°C until use.

### Treatments of *C*. *jejuni* conditioned culture supernatant

Conditioned culture supernatants were incubated (3 h, 37°C) with polymyxin B (100 μg ml^-1^), proteinase K (100 μg ml^-1^), DNase (200 U ml^-1^) or RNase (200 U ml^-1^), followed by an enzyme inactivation step (5 min, 95°C). Mock-treated culture supernatant was treated identical without the addition of enzymes or polymyxin B. None of the treatments by themselves had any effect on NF-κB activity in HeLa 57A cells. All enzymes and polymyxin B were purchased from Sigma-Aldrich. Heat-treatment of conditioned culture supernatant was performed in a 95°C waterbath for 10 min. Size-fractionation of conditioned culture supernatant was done using Pierce protein concentrators of different pore-size (PES 3K MWCO, PES 30K MWCO, PES 100K MWCO, ThermoFisher Scientific) according to manufacturer’s protocol. Sodium (meta)periodate (10 mM, Sigma-Aldrich) treatment of conditioned culture supernatant was performed for 1 h at 20°C. As a control TNFα (0.5 μg ml^-1^) was treated with sodium (meta)periodate under identical conditions. Periodate itself did not influence NF-κB activity in HeLa 57A cells.

### Stimulation of HeLa 57A reporter cells, NF-κB luciferase reporter assay and IL-8 ELISA

Twenty-four hours after seeding of HeLa 57A cells into 96-well plates, media was replaced with 100 μl of DMEM followed by the addition of either 10 μl of *Campylobacter* conditioned supernatant, 2 μl of the various products of the heptose biosynthesis pathway, or ligands for TLRs or NOD1/2 (Pam_3_CSK_4_ at 100 ng ml^-1^, FSL-1 at 100 ng ml^-1^, Poly I:C at 50 μg ml^-1^, CL-97 at 5 μg ml^-1^, ODN2006 at 5 μM, C12-iE-DAP at 1 μg ml^-1^, L18-MDP at 1 μg ml^-1^, LPS from *N*. *meningitidis* at 100 ng ml^-1^ (purified as described in [31]) and flagellin from *Salmonella enterica* serovar Enteritidis strain 706 at 500 ng ml^-1^ (purified as described in [32]). After stimulation for 5 h at 37°C, the medium was carefully removed and cells were lysed by adding 50 μl Reporter Lysis Buffer (Promega) and frozen at -80°C for a minimum of 45 min. After thawing, 15 μl of lysate was mixed with 37 μl of Luciferase Assay Reagent (Promega) and luciferase activity was measured with a TriStar2 luminometer (Berthold). NF-κB dependent luciferase activity is indicated in Relative Light Units (RLU) and are presented as fold increase in stimulated versus unstimulated cells. To assess the concentration of IL-8, supernatants were collected after 18 h of stimulation and examined using the Human IL-8/CXCL8 DuoSet ELISA DY208 (R&D Systems), according to the manufacturer’s protocol.

### Generation of *C*. *jejuni* mutants

*C*. *jejuni* mutants were generated as described previously [16]. In short, target genes and their flanking regions were PCR amplified from chromosomal DNA from strain 81116 using the primers 5’-actggagtaaagaatggtggca-3’ and 5’-gagtgtcgcaaacctaaagca-3’ (for *hldE*), 5’-cgcgaacaatcacaccaagt-3’ and 5’-gcttcttgtctacccgagga-3’ (for *gmhB*), or 5’-tgcaaaaacttctgtataggaacga-3’ and 5’-acttgcaaagcgtctatggaa-3’ (for *waaC*), ligated into pJET1.2 and transformed into *E*. *coli* DH5α. Resulting plasmids were used as template in an outward PCR with primers 5’-cgcggatccaagtgtaattatccaccataaaatcc-3’ and 5’-cgcggatccgttgaattaatcgactttgaagaagg-3’ (for *hldE*), 5’-cgcggatcctttttatctatattaatcacaccgtctctatc-3’ and 5’-cgcggatcctggcactttgattttagtaaatgaag-3’ (for *hldE*), or 5’-ccggatccgtgcaacaccaagctaccg-3’ and 5’-ccggatccgtgctgataaacgaacaattgc-3’ (for *waaC*) to inactivate the corresponding genes. Unique BamHI restriction sites were introduced to enable insertion of the chloramphenicol cassette from pAV35. Gene inactivation constructs were verified by sequencing. The plasmids were introduced individually via natural transformation into *C*. *jejuni* using chloramphenicol (20 μg ml^-1^) selection. All gene disruptions in *C*. *jejuni* were confirmed via PCR. Deletion mutants 81116Δ*hldE* and 81116Δ*gmhB* were complemented by shuttle vectors expressing the corresponding intact genes from a constitutive MetK promotor [33]. Target genes were PCR amplified from chromosomal DNA from strain 81116 using the primers 5’-ccgagctcaaaaggaagtaaatgcttgagtttttaagtcagc-3’ and 5’-ccccgcggtcattttttatccttaatcttttctatg-3’ (for *hldE*) and 5’-ccgagctcaaaaggaagtaaatgtttattttctttttaaaaaagctatatta-3’ and 5’-ccccgcggttaaatgtccttttctttaaaaaagc-3’ (for *gmhB*), ligated into pMA1 [33] using SacI and SacII restriction sites and transformed into *E*. *coli* S17-1 λpir using kanamycin (25 μg ml^-1^) selection. Resulting plasmids were verified by sequencing and transformed to the corresponding *C*. *jejuni* deletion mutant via conjugation [33].

### Electrophoretic analysis of LOS

LOS molecules were separated by electrophoresis of proteinase K-treated bacterial pellets on a 16% tricine SDS-PAGE gel [34]. LOS bands were silver-stained according to [35].

### Construction, expression and purification of recombinant His-tagged proteins GmhA, HldE and GmhB of wild-type *C*. *jejuni* 81116

*Campylobacter jejuni* 81116 *gmhA*, *hldE* and *gmhB* genes were amplified with primers 5’-GCTTAATTAAATGATAAATTTAGTGGAAAAAGAATG-3’ and 5’- GCACCGGTAAAACCTTCATCTATAATCTGGC-3’ (*gmhA*), 5’-GCTTAATTAAATGCTTGAGTTTTTAAGTCAGC-3’ and 5’- GCACCGGTTTTTTTATCCTTAATCTTTTCTATGATC-3’ (*hldE*), and 5’-GCTTAATTAAATGTTTATTTTCTTTTTAAAAAAGCTATAT-3’ and 5’-GCACCGGTAATGTCCTTTTCTTTAAAAAAGCTC-3’ (*gmhB*), and cloned into pBac-6xH, an in-house derivative of pET101 that expresses genes from an T7 promotor in frame with an C-terminal 6xHIS tag. Gene expression was induced in early-log growing bacterial cultures (LB broth supplemented with 20 μM ZnSO_4_-7H_2_O) with 0.5 mM isopropyl-beta-D-thiogalactopyranoside (IPTG, Thermo Scientific) and incubated at 30°C for 5 h (shaking at 160 rpm). Bacterial pellets were lysed in resuspension buffer (50 mM NaH_2_PO_4_, 300 mM NaCl, 10 mM imidazole, pH 8.0), 1 mg ml^-1^ lysozyme, protease inhibitor (EDTA-free, pH 8.0, Roche) for 45 min at 4°C followed by sonication. Cleared lysates were prepared by centrifugation (10,000 × g, 30 min, 4°C) and incubated with nickel-coated beads (nickel–nitrilotriacetic acid [Ni–NTA] Agarose, Thermo Scientific) for 1 h at 4°C with end-of-end rotation. NTA beads were washed with 20 ml of washing buffer (50 mM NaH_2_PO_4_, 300 mM NaCl, 50 mM imidazole, pH 8.0). Proteins were eluted from the NTA-agarose using elution buffer (50 mM NaH_2_PO_4_, 300 mM NaCl, 250 mM imidazole, pH 8.0) and dialyzed overnight in 20 mM HEPES buffer, 20 mM KCl, 1 mM DTT, pH 7.0 at 4°C buffer. Protein concentration were determined using Pierce BCA Protein Assay Kit (Thermo Scientific) and were stored in 50% glycerol at -20°C.

### *In vitro* synthesized *C*. *jejuni* heptose metabolites

ADP-heptose was enzymatically synthesized in the following reaction: 20 mM HEPES buffer (pH 6.5), 20 mM KCl, 10 mM MgCl_2_, 2 mM Sedoheptulose-7-phosphate (Sigma), 6 mM ATP, 2 μg recombinant GmhA, 4 μg recombinant HldE and 2 μg recombinant GmhB in a total volume of 100 μl. Synthesis was performed at 37°C for 18 h and stopped by incubating at 98°C for 10 min. ADP-heptose was subsequently stored at -20°C. Where appropriate, enzymes were replaced with 10 mM HEPES buffer (pH 7.0), 10 mM KCl, 0.5 mM DTT, 50% glycerol. As mock stimulation, GmhA, HldE and GmhB enzymes were heat-inactivated at 98°C for 10 min prior to adding to the synthesis reactions, which were subsequently performed under identical conditions as described above.

### CRISPR-Cas9 mediated inactivation of Alpk1 in HeLa 57A cells

Two different gRNAs targeting exon 3, which includes the start codon of human *ALPK1*, were designed. Two complimentary oligos for each gRNA (oligos for gRNA-1: 5’-CACCGCATCATGAATAATCAAAAAG-3’ and 5’-AAACCTTTTTGATTATTCATGATGC-3’; oligos for gRNA-2: 5’-CACCGTCTAGGGTGATCAATTACCT-3’ and 5’-AAACAGGTAATTGATCACCCTAGAC-3’) were annealed and ligated into the vector pSpCas9(BB)-2A-GFP [36], which expresses a Cas9 from *S*. *pyogenes* fused to 2A-EGFP and contains a cloning backbone for sgRNA. pSpCas9(BB)-2A-GFP (PX458) was a gift from Feng Zhang (Addgene plasmid # 48138; http://n2t.net/addgene:48138; RRID:Addgene_48138). HeLa 57A cells were grown in a 6-well plate in DMEM with 10% FCS. After 24 h cells were transfected at 70% confluency with 1000 ng of pSpCas9(BB)-2A-GFP-gRNA-1 and pSpCas9(BB)-2A-GFP -gRNA-2 plasmid (2000 ng in total) using Fugene HD (Promega) according to the manufacturer’s instructions. At 24 h after transfection culture medium was refreshed. At 48 h after transfection cells were washed with DPBS (Sigma), detached using trypsin, resuspended in DMEM with 10% FCS and sorted for GFP expression by FACS. Cells with the strongest GFP expression were seeded individually (single-cell) in a well of a 96-well plate containing growth medium consisting of: 20% FCS, 30% conditioned medium (cell free supernatant of a 60 h HeLa 57A culture at 80% confluency), 50% fresh DMEM, 10,000 units ml^-1^ penicillin and streptomycin (Jena Bioscience) and 2.5 μg ml^-1^ Amphotericin B (Sigma). Independent clones were stimulated with sterile conditioned culture supernatant of *C*. *jejuni* or TNF-α as positive control. Clones that showed no activation of NF-κB following stimulation with *C*. *jejuni* supernatant, but retained NF-κB activity upon TNF-α stimulation were selected for further expansion. To exclude off-target effects, clones were seeded in a 96-well plate, transfected with (500 ng) pTracer CMV-2 carrying human Alpk1 using FuGENE 6 reagent (Promega) and after 48 h stimulated with *C*. *jejuni* supernatants. Two clones (A and B) that regained sensitivity for ADP-heptose were frozen for further use. To identify the genetic mutations within the ALPK1 gene, genomic DNA of HeLa 57A wildtype cells, ALPK1^-/-^ clone A and B was isolated and used as template in a PCR with primers ALPK1-seqF 5’-GAGTCTCACAGATAATTGATGA-3’ and ALPK1-seqR 5’-AATAGGGCAGAGATAAGAAC-3’. Resulting PCR products were cloned into pJET1.2/Blunt (Thermo Fisher Scientific), individual colonies were cultured, plasmid DNA was isolated and the PCR inserts were Sanger-sequenced (Macrogen).

### Microarray gene expression analysis

HeLa 57A cells grown in a 6-well plate for 18 h were stimulated with 80 μl of *C*. *jejuni* conditioned culture medium (sterilized through a 3kDa filter) without changing media. After 2 h of incubation, RNA was isolated using Trizol treatment (ThermoFisher Scientific) according to manufacturer’s protocol followed by purification using a RNeasy column (QIAGEN) with on column DNase treatment. Eluted RNA was aliquoted and used for the RNA array. The purity and concentration of the RNA was analyzed using a BioAnalyzer (Agilent). The microarray experiment was performed 4 times, as a direct comparison between treated and control HeLa 57A cells. Opposite labeling of two of four sample sets generated a balanced dye-swap design. Microarrays used were human whole genome gene expression microarrays V2 (Agilent, Belgium) representing 34127 *H*. *sapiens* 60-mer probes in a 4x44K layout. Probe sequences from this array were re-annotated by BLAST-searching against genomebuild version 76_38 at EMSEMBL. cDNA synthesis, cRNA amplification, labeling, quantification, quality control and fragmentation were performed with an automated system (Caliper Life Sciences NV/SA, Belgium), starting with 3 ug of total RNA from each sample, as previously described in detail [37]. Microarray hybridization and washing was done with a HS4800PRO system with QuadChambers (Tecan, Benelux) using 1200 ng, 1.5–2% Cy5/Cy3 labeled cRNA per channel as described [37]. Slides were scanned on an Agilent G2565BA scanner at 100% laser power, 30% PMT. After automated data extraction using Imagene 8.0 (BioDiscovery), Loess normalization was performed [38] on mean spot-intensities. Data were further analyzed by MAANOVA [39], modeling sample, array and dye effects in a fixed effect analysis. P-values were determined by a permutation F2-test, in which residuals were shuffled 10,000 times globally. Gene probes with *p*<0.05 after family wise error correction (FWER) were considered significantly changed. In cases of multiple probes per gene, the values from the most 3’ probe were used. A fold change cutoff of 2 was used. The microarray results have been deposited in NCBI’s Gene Expression Omnibus and are accessible through GEO Series accession number GSE70610 (http://www.ncbi.nlm.nih.gov/geo/query/acc.cgi?acc=GSE70610) [40].

### Stimulation of HT29-MTX cells

HT29-MTX cells were split into 24-well plates and grow for 24 h prior to addition of 5 μl *in vitro* synthesized ADP-heptose and stimulation for 3 h. As a control, cells were untreated or treated with 5 μl mock-synthesized ADP-heptose. For the infection experiments with viable bacteria, *C*. *jejuni* 81116 or 81116Δ*hldE* were grown for 17 h, washed gently in 1 ml DMEM without FCS to remove all traces of culture supernatant. Bacteria were added to HT29-MTX cells, grown for 24 h in 24-well plates with culture medium replaced with DMEM without FCS just prior to infection, at a multiplicity of infection (MOI) of 200 for 10 h at 37°C and 5% CO_2_.

### Real time RT-PCR

For the *C*. *jejuni* infection experiments, total RNA from HT29-MTX cells, cultured in 24-well plates as described above, was extracted using RNA-Bee (Tel-Test, Inc.) according to the manufacturer’s protocol. RNA from primary enterocytes (Lonza), cultured in 48-well plates for 6 days, was isolated using Trizol (ThermoFisher Scientific) according to manufacturer’s protocol. RNA was eluted in diethyl pyrocarbonate (DEPC) water, analyzed for quality and concentration spectrophotometrically by measuring the absorbance at 260 nm and 280 mm (Nanodrop, Thermo Scientific, Waltham, MA, USA) and stored at -80°C until use. Synthesis of first strand cDNA was performed with RevertAid First Strand cDNA Synthesis Kit (Thermo Fisher) according to the manufacturer’s instructions. cDNA was used as template in quantitative polymerase chain (qPCR) experiments with TaqMan Fast Advanced Master Mix (ThermoFisher Scientific) and TaqMan Gene Expression Assays (*GAPDH*: Hs02758991_g1, *CXCL8*: Hs00174103_m1, *CXCL2*: Hs00601975_m1, *PTGS2*: Hs00153133_m1, *TNFAIP2*: Hs00969305_m1, *ALPK1*: Hs01567926_m1, *TIFA*: Hs00385268_m1 (ThermoFisher Scientific)) in a LightCycler 480 Real-Time PCR System (Roche) according to the manufacturer’s instructions. mRNA levels were calculated by subtracting the corresponding C_t_ values obtained for samples before (1) and after (2) treatment using the following formula: (1) ΔC_t control_ = C_t target gene control_−C_t Gapdh control_, (2) ΔC_t target gene treat_−C_t Gapdh treated_. The fold change in mRNA was determined by: Fold change = 2^(ΔCt(treated)-(ΔCt(control))^ [22]. Presented results are from four individual assays performed.

### Statistical analysis

Results were analyzed using GraphPad Prism 8 software. Where appropriate significance was calculated using a non-paired Student t-test. Significance was annotated with asterisk; P value 0.01–0.05 = *, 0.001–0.01 = **, 0.001 = ***, not significant = ns.

## Supporting information

S1 Table Bacterial strains used in this study. (DOCX)

S2 Table Results of the microarray analysis of HeLa 57A cells stimulated with *C*. *jejuni*-released ADP-heptose. Shown are all genes that are either up- or downregulated with a statistical significance of p < 0.05.
(XLSX)

S1 Fig *C*. *jejuni* 81116 releases a novel activator of NF-κB into the culture supernatant at stationary growth phase. HeLa 57A cells were stimulated with sterile *C*. *jejuni* strain 81116-conditioned culture supernatant or *C*. *jejuni* strain 81116 cells disrupted via sonification at timepoints 16, 20, 24, 40, 44 and 48 h after the start of culturing. *C*. *jejuni* was cultured in HI broth. NF-κB activation is measured as relative luciferase units and presented as fold increase in stimulated versus unstimulated cells, and depicted on the left Y-axis. Bacterial growth was assessed by measuring the optical density at 550 nm at each timepoint and depicted on the right Y-axis. Values represent the mean ± SEM of three independent experiments performed in duplicate.
(TIF)

S2 Fig Multiple strains of *C*. *jejuni* release a novel activator of NF-κB into the culture supernatant independent of growth media. HeLa 57A cells were stimulated with sterile *C*. *jejuni*-conditioned culture supernatant or *C*. *jejuni* cells disrupted via sonification (bacterial lysates) of strains 81116, 11168, 81–176, GB18 or 108 at timepoints 16, 20, 24, 40, 44 and 48 h after the start of culturing. *C*. *jejuni* was cultured in HI broth, MH broth or Bolton broth. NF-κB activation is measured as relative luciferase units and presented as fold increase in stimulated versus unstimulated cells. Values represent the mean ± SEM of three independent experiments performed in duplicate.
(TIF)

S3 Fig Expression of *ALPK1* and *TIFA* in HeLa 57A and HT29-MTX cells. (A) PCR products specific for *ALPK1* and *TIFA* were obtained by RT-PCR with isolated total RNA from HeLa 57A and HT29-MTX, conversed to cDNA, as template and separated on a 2% agarose gel. Control PCR reactions were performed without reverse transcriptase (-RT) to test for chromosomal DNA contamination. (B) Relative expression of *ALPK1* and *TIFA* in HeLa 57A and HT29-MTX cells, cultured for 24 h, as compared to the housekeeping gene *GAPDH*.
(TIF)

S4 Fig *C*. *jejuni*-derived ADP-heptose-mediated NF-κB activation is dependent on ALPK1. (A) Schematic overview of the CRISPR/Cas9 deletion strategy, showing the locations of the two sgRNAs targeting the *ALPK1* exon 3. (B) Sequence analysis of the *ALPK1* exon 3 from wild type HeLa 57A cells and to HeLa 57A ALPK1^-/-^ Clone B. (C) Wild type HeLa 57A cells or (D) HeLa 57A cells deficient in ALPK1 (HeLa 57A ALPK1^-/-^, Clone B) were transfected with an empty expression plasmid or transfected with a plasmid expressing human *ALPK1*, and subsequently stimulated for 5 h with sterile conditioned culture medium of wild type *C*. *jejuni* 81116, *C*. *jejuni* 81116Δ*hldE*, mock ADP-heptose of ADP-heptose derived from *in vitro* synthesis using *C*. *jejuni* enzymes. NF-κB activation was measured as relative luciferase units and presented as fold increase in stimulated versus unstimulated cells. Results are the mean ± SEM of three independent experiments performed in duplicate. *p < 0.05, **p < 0.01, and ***p < 0.001.
(TIF)
