# Supplementary material for: The ALPK1 pathway drives the inflammatory response to Campylobacter jejuni in human intestinal epithelial cells
Source: PLoS Pathog. 2021 Aug 2;17(8):e1009787. doi: 10.1371/journal.ppat.1009787 (PMC8360561; doi:10.1371/journal.ppat.1009787)
Supplement: S1 Table — (DOCX) [file ppat.1009787.s001.docx]

**Supplemental Table 1.** Bacterial strains used in this study.

| **Strain name** | **Description** | **Reference** |
| --- | --- | --- |
| *C. jejuni* 81116 | Human isolate | (1) |
| *C. jejuni* 11168 | Human isolate, NCTC11168 | (2) |
| *C. jejuni* 81-176 | Human isolate | (3) |
| *C. jejuni* GB18 | Isolated from a patient with Guillain Barré Syndrome | (4) |
| *C. jejuni* 108 | Intestinal isolate from patient with bacteremia | (5) |
| *C. lari* subsp*. lari* | LMG 14338 |  |
| *C. fetus* subsp. *fetus* | 04/554 |  |
| *C. coli* H1 | In-house chicken isolate |  |
| *C. mucosalis* | LMG 7794 |  |
| *C. sputorum* subsp. *sputorum* | LMG 6617 |  |
| *C. helveticus* | 30563 |  |
| *E. coli*  *S*. Typhimurium  *Klebsiella* sp.  *Y. pseudotuberculosis*  *C. freundii*  *L. monocytogenes*  *B. cereus*  *C. albicans*  *C. jejuni* 81116Δ*hldE* | In-house clinical isolate  In-house clinical isolate  In-house clinical isolate  In-house clinical isolate  In-house clinical isolate  In-house clinical isolate  In-house clinical isolate  In-house clinical isolate  *C. jejuni* 81116 in which the *hldE* gene has been replaced by a chloramphenicol resistance cassette | This study |
| *C. jejuni* 81116Δ*hldE* + pHldE  *C. jejuni* 81116Δ*gmhB*  *C. jejuni* 81116Δ*gmhB* + pGmhB  *C. jejuni* 81116Δ*waaF*  *C. jejuni* 81116Δ*waaC* | *C. jejuni* 81116Δ*hldE* complemented with a plasmid expressing the *hldE* gene from the constitutive MetK promotor  *C. jejuni* 81116 in which the *gmhB* gene has been replaced by a chloramphenicol resistance cassette  *C. jejuni* 81116Δ*gmhB* complemented with a plasmid expressing the *gmhB* gene from the constitutive MetK promotor  *C. jejuni* 81116 in which the *waaF* gene has been replaced by a chloramphenicol resistance cassette  *C. jejuni* 81116 in which the *waaC* gene has been replaced by a chloramphenicol resistance cassette | This study  This study  This study  This study  This study |
|  |  |  |

**Supplementary References**

1. Newell DG, McBride H, Dolby JM. Investigations on the role of flagella in the colonization of infant mice with *Campylobacter jejuni* and attachment of *Campylobacter* *jejuni* to human epithelial cell lines. J Hyg (Lond). 1985;95(2):217-27.

2. Parkhill J, Wren BW, Mungall K, Ketley JM, Churcher C, Basham D, et al. The genome sequence of the food-borne pathogen *Campylobacter* *jejuni* reveals hypervariable sequences. Nature. 2000;403(6770):665-8.

3. Black RE, Levine MM, Clements ML, Hughes TP, Blaser MJ. Experimental *Campylobacter jejuni* Infection in Humans. The Journal of Infectious Diseases. 1988;157(3):472-9.

4. Ang CW, De Klerk MA, Endtz HP, Jacobs BC, Laman JD, van der Meché FG, et al. Guillain-Barré syndrome- and Miller Fisher syndrome-associated *Campylobacter jejuni* lipopolysaccharides induce anti-GM1 and anti-GQ1b Antibodies in rabbits. Infect Immun. 2001;69(4):2462-9.

5. van Alphen LB, Bleumink-Pluym NM, Rochat KD, van Balkom BW, Wösten MM, van Putten JP. Active migration into the subcellular space precedes *Campylobacter jejuni* invasion of epithelial cells. Cell Microbiol. 2008;10(1):53-66.
