# Supplementary figures and images for: The ALPK1 pathway drives the inflammatory response to Campylobacter jejuni in human intestinal epithelial cells
Source: PLoS Pathog. 2021 Aug 2;17(8):e1009787. doi: 10.1371/journal.ppat.1009787 (PMC8360561; doi:10.1371/journal.ppat.1009787)

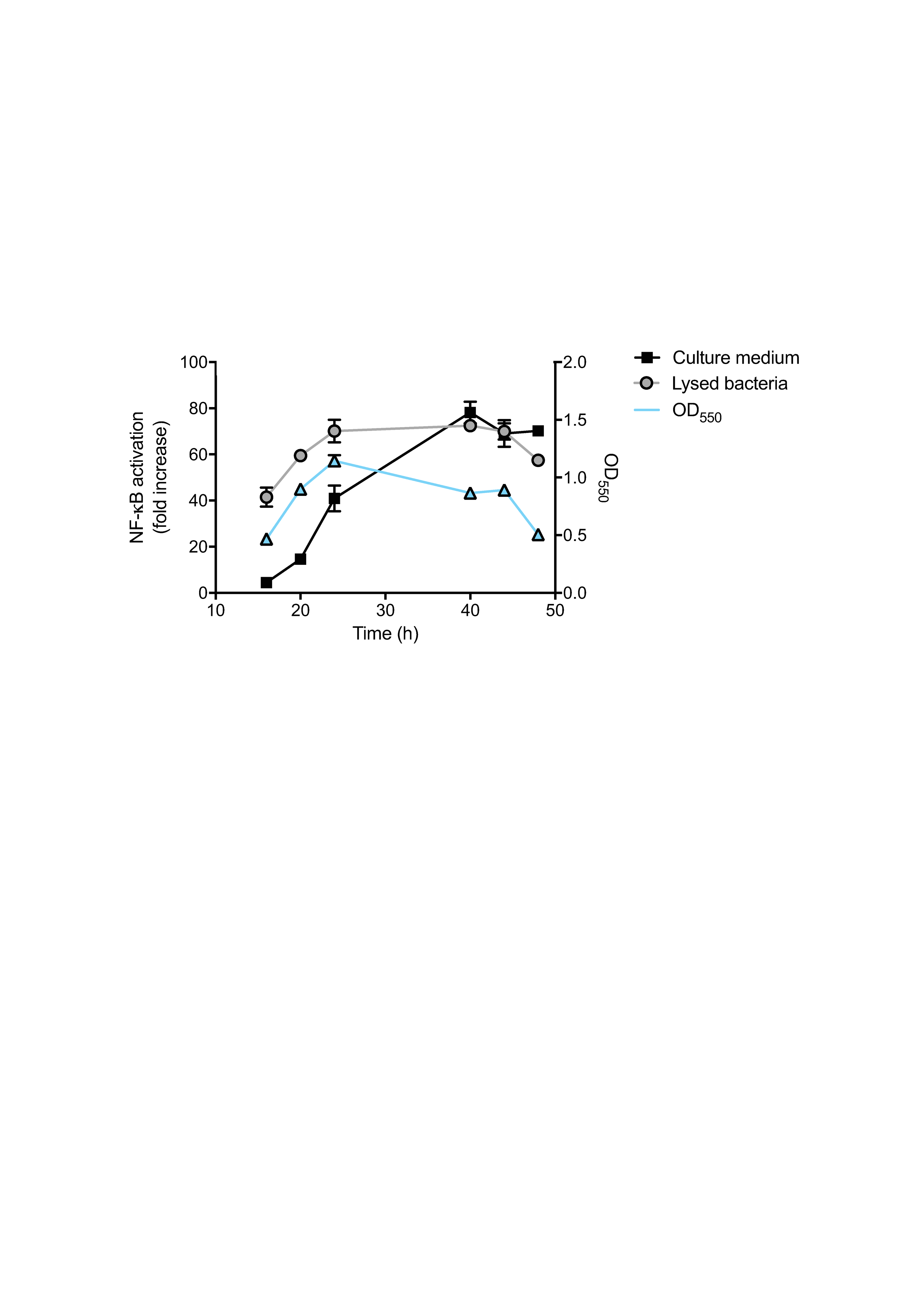

Supplement: S1 Fig — HeLa 57A cells were stimulated with sterile C. jejuni strain 81116-conditioned culture supernatant or C. jejuni strain 81116 cells disrupted via sonification at timepoints 16, 20, 24, 40, 44 and 48 h after the start of culturing. C. jejuni was cultured in HI broth. NF-κB activation is measured as relative luciferase units and presented as fold increase in stimulated versus unstimulated cells, and depicted on the left Y-axis. Bacterial growth was assessed by measuring the optical density at 550 nm at each timepoint and depicted on the right Y-axis. Values represent the mean ± SEM of three independent experiments performed in duplicate. (TIF) [file ppat.1009787.s003.tif]

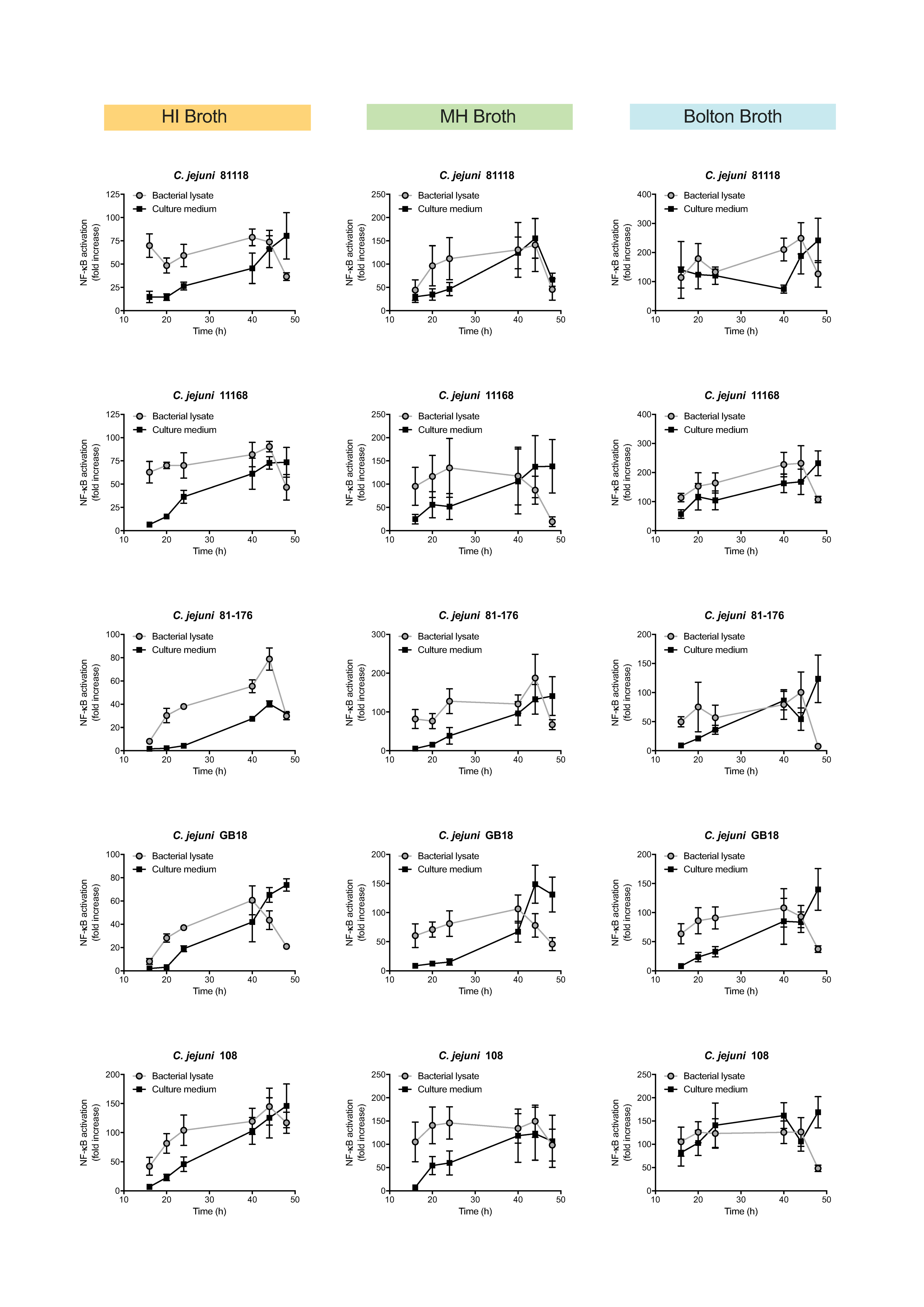

Supplement: S2 Fig — HeLa 57A cells were stimulated with sterile C. jejuni-conditioned culture supernatant or C. jejuni cells disrupted via sonification (bacterial lysates) of strains 81116, 11168, 81–176, GB18 or 108 at timepoints 16, 20, 24, 40, 44 and 48 h after the start of culturing. C. jejuni was cultured in HI broth, MH broth or Bolton broth. NF-κB activation is measured as relative luciferase units and presented as fold increase in stimulated versus unstimulated cells. Values represent the mean ± SEM of three independent experiments performed in duplicate. (TIF) [file ppat.1009787.s004.tif]

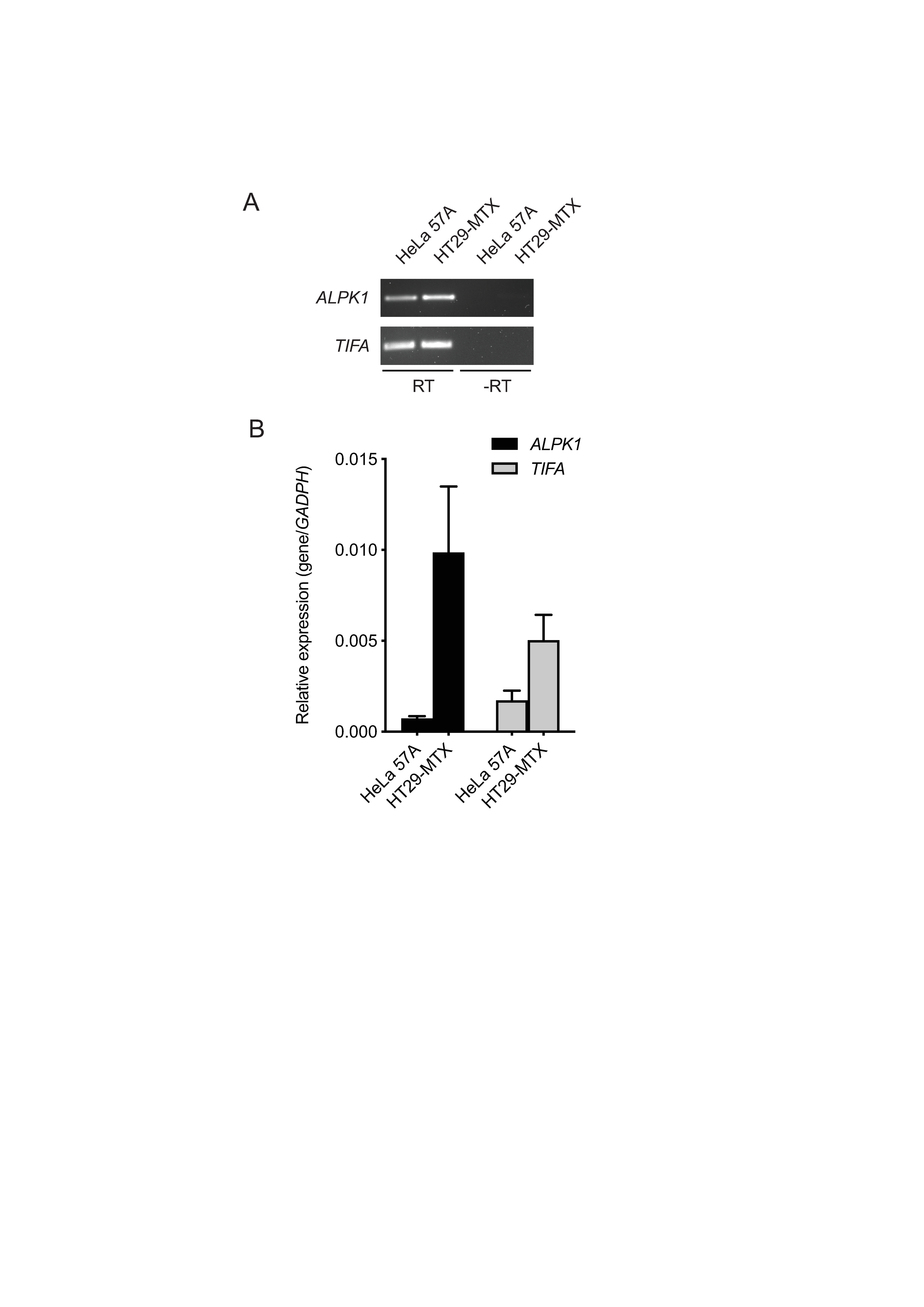

Supplement: S3 Fig — (A) PCR products specific for ALPK1 and TIFA were obtained by RT-PCR with isolated total RNA from HeLa 57A and HT29-MTX, conversed to cDNA, as template and separated on a 2% agarose gel. Control PCR reactions were performed without reverse transcriptase (-RT) to test for chromosomal DNA contamination. (B) Relative expression of ALPK1 and TIFA in HeLa 57A and HT29-MTX cells, cultured for 24 h, as compared to the housekeeping gene GAPDH. (TIF) [file ppat.1009787.s005.tif]

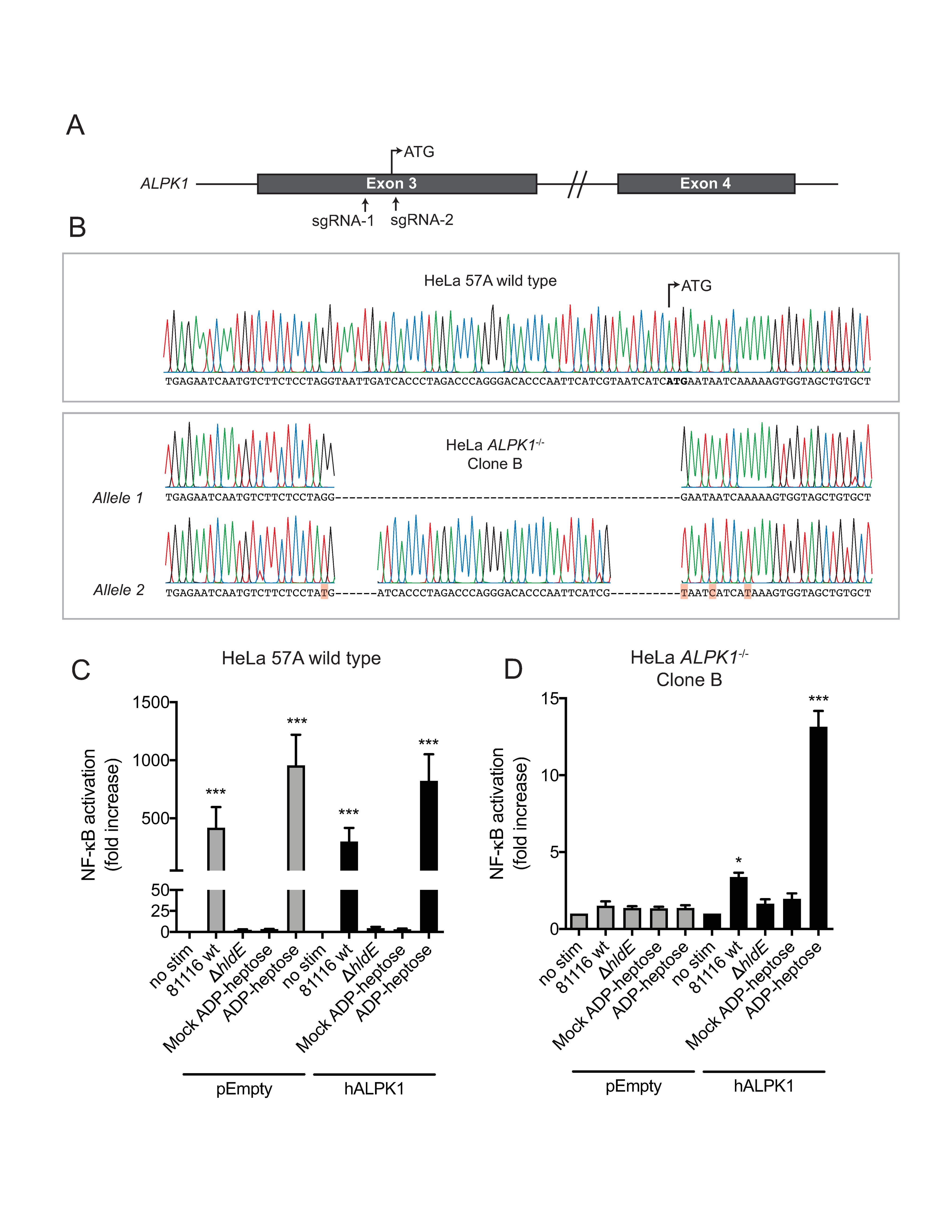

Supplement: S4 Fig — (A) Schematic overview of the CRISPR/Cas9 deletion strategy, showing the locations of the two sgRNAs targeting the ALPK1 exon 3. (B) Sequence analysis of the ALPK1 exon 3 from wild type HeLa 57A cells and to HeLa 57A ALPK1-/- Clone B. (C) Wild type HeLa 57A cells or (D) HeLa 57A cells deficient in ALPK1 (HeLa 57A ALPK1-/-, Clone B) were transfected with an empty expression plasmid or transfected with a plasmid expressing human ALPK1, and subsequently stimulated for 5 h with sterile conditioned culture medium of wild type C. jejuni 81116, C. jejuni 81116ΔhldE, mock ADP-heptose of ADP-heptose derived from in vitro synthesis using C. jejuni enzymes. NF-κB activation was measured as relative luciferase units and presented as fold increase in stimulated versus unstimulated cells. Results are the mean ± SEM of three independent experiments performed in duplicate. *p < 0.05, **p < 0.01, and ***p < 0.001. (TIF) [file ppat.1009787.s006.tif]
